# Photodynamic Therapy Activity of New Porphyrin-Xylan-Coated Silica Nanoparticles in Human Colorectal Cancer

**DOI:** 10.3390/cancers11101474

**Published:** 2019-09-30

**Authors:** Ludovic Bretin, Aline Pinon, Soukaina Bouramtane, Catherine Ouk, Laurence Richard, Marie-Laure Perrin, Alain Chaunavel, Claire Carrion, Frédérique Bregier, Vincent Sol, Vincent Chaleix, David Yannick Leger, Bertrand Liagre

**Affiliations:** 1 Laboratoire PEIRENE EA 7500, Faculté de Pharmacie, Université de Limoges 2, Rue du Docteur Raymond Marcland, 87025 Limoges Cedex, France; ludovic.bretin@unilim.fr (L.B.); aline.pinon@unilim.fr (A.P.); david.leger@unilim.fr (D.Y.L.); 2 Laboratoire PEIRENE EA 7500, Faculté des Sciences & Techniques, Université de Limoges 123, Avenue Albert Thomas, 87060 Limoges Cedex, France; soukaina.bouramtane@unilim.fr (S.B.); frederique.bregier@unilim.fr (F.B.); vincent.sol@unilim.fr (V.S.); vincent.chaleix@unilim.fr (V.C.); 3 BISCEm Pôle Cytométrie en flux/Microscopie, Université de Limoges 2, Rue du Docteur Raymond Marcland, 87025 Limoges Cedex, France; catherine.ouk@unilim.fr (C.O.); claire.carrion@unilim.fr (C.C.); 4 Service d’Anatomie Pathologique, Centre Hospitalier Universitaire de Limoges 2, Avenue Martin Luther King, 87042 Limoges Cedex, France; laurence.richard@unilim.fr (L.R.); alain.chaunavel@chu-limoges.fr (A.C.); 5 Laboratoire Bio EM XLIM UMR CNRS 7252, Faculté de Médecine, Université de Limoges 2, Rue du Docteur Raymond Marcland, 87025 Limoges Cedex, France; marie-laure.perrin@unilim.fr

**Keywords:** anticancer drug, porphyrin, silica nanoparticles, drug delivery, photodynamic therapy

## Abstract

Photodynamic therapy (PDT) using porphyrins has been approved for treatment of several solid tumors due to the generation of cytotoxic reactive oxygen species (ROS). However, low physiological solubility and lack of selectivity towards tumor sites are the main limitations of their clinical use. Nanoparticles are able to spontaneously accumulate in solid tumors through an enhanced permeability and retention (EPR) effect due to leaky vasculature, poor lymphatic drainage, and increased vessel permeability. Herein, we proved the added value of nanoparticle vectorization on anticancer efficacy and tumor-targeting by 5-(4-hydroxyphenyl)-10,15,20-triphenylporphyrin (TPPOH). Using 80 nm silica nanoparticles (SNPs) coated with xylan-TPPOH conjugate (TPPOH-X), we first showed very significant phototoxic effects of TPPOH-X SNPs mediated by post-PDT ROS generation and stronger cell uptake in human colorectal cancer cell lines compared to free TPPOH. Additionally, we demonstrated apoptotic cell death induced by TPPOH-X SNPs-PDT and the interest of autophagy inhibition to increase anticancer efficacy. Finally, we highlighted in vivo, without toxicity, elevated anticancer efficacy of TPPOH-X SNPs through improvement of tumor-targeting compared to a free TPPOH protocol. Our work demonstrated for the first time the strong anticancer efficacy of TPPOH in vitro and in vivo and the merit of SNPs vectorization.

## 1. Introduction

In 2018, colorectal cancer (CRC) was the third most common cancer with 1.8 million cases globally, and the second leading cause of death for oncological reasons with 862,000 deaths [1]. The conventional treatment options for patients with CRC are surgery, chemotherapy, and/or radiotherapy, which unfortunately has many side effects and long recovery periods. Over the past decade, significant progress in CRC treatment has been achieved through the development of novel drugs and treatment protocols. However, the increasing resistance of tumor cells toward these novel drugs and persistent side effects due to toxicity on healthy tissues make it imperative to find other methods of CRC therapy [2,3,4,5,6]. 

Photodynamic therapy (PDT), an alternative cancer treatment, appears to be a promising option [7]. The molecular mechanism of PDT involves simultaneous interaction between a photosensitizer (PS), a light source with an appropriate wavelength, and molecular oxygen. Relative to traditional therapies, PDT has several advantages including non-invasive therapy, non-cytotoxic molecules without light activation, and site-specific light treatment which decreases the side effects, thus accelerating the healing process [8,9]. PDT is based on the generation of reactive oxygen species (ROS) which mediate cellular toxicity. Upon light irradiation, the PS is activated from a ground to an excited state. The excited PS is very unstable and loses its excess energy either directly or via the excited triplet state. The excited triplet state is generated by intersystem crossing. In this long-lived excited triplet state, the PS slowly returns to the ground state through type I or type II photochemical reactions. In the first reaction, the excited PS reacts with a biological substrate via hydrogen or electron transfer, producing free radical species. These species can react with molecular oxygen producing ROS such as the superoxide radical anion (O_2_^•−^) and hydrogen peroxide (H_2_O_2_). In the second reaction, the excited PS transfers its energy directly to molecular oxygen to form singlet oxygen (^1^O_2_). These highly cytotoxic ROS can oxidize a variety of biomolecules, induce an acute stress response, and trigger a series of redox signaling pathways, leading to cell death frequently through apoptosis [10,11,12,13]. At present, tetrapyrrole compounds such as porphyrins, chlorins, bacteriochlorins, and phthalocyanines are the most commonly used PS in PDT [14]. However, the main disadvantages of these PS are their poor solubility in water, which limits intravenous administration, their low photo-physical properties due to the aggregation of PS, and their poor tumor selectivity limiting their use in clinical protocols. 

In order to increase the selectivity and bioavailability of PS, new drug delivery systems are emerging. Nanotechnology using nanocarriers appears to be the most promising strategy. Nanoparticles (NPs) are able to spontaneously accumulate in solid tumors through passive targeting: the enhanced permeability and retention (EPR) effect occurs due to a combination of leaky vasculature, poor lymphatic drainage, and increased vessel permeability. Vectorizing NPs through encapsulation or attachment of PS not only enhances tumor-targeting through the EPR effect but also increases the hydrophilicity and tissue lifetime of PS [15,16,17,18]. Recently, various nanocarriers have been developed using organic and inorganic strategies. Organic drug delivery systems suffer from intrinsic instability and low drug-loading capacity/efficiency, restricting their further clinical potential. However, inorganic NPs show high chemical stability and resistance to corrosion under physiological conditions. Among these inorganic NPs, silica nanoparticles (SNPs) have been recognized as promising nanocarriers for PDT. SNPs have numerous advantages such as their easy synthesis, stability, controllable size, modifiable surface potential and easy functionalization. One of the major issues of inorganic NPs is biocompatibility. SNPs are one of the most biocompatible materials, thereby increasing interest in their use for medical applications [19,20,21,22].

One of the biggest challenges in controlled drug delivery systems is blood circulation half-life due to mononuclear phagocyte sequestration. To delay opsonization and increase nanocarrier lifetimes in the bloodstream, coating NPs with hydrophilic groups appears to be a promising strategy. This strategy involves grafting long chain polymers such as polyethylene glycol or polysaccharides on NPs. These polymers create a protective hydrophilic layer around the NPs which prevents the binding of opsonins via steric repulsion forces, thus delaying opsonization and phagocytosis of NPs [23,24,25]. Xylan, a hemicellulose, is defined as a group of cell wall polysaccharides. Xylan is a natural, biodegradable, and non-toxic biomaterial which has been reported to stabilize magnetic NPs in biological media with improvement of their biocompatibility and biodistribution [26,27]. Xylan has been also demonstrated to play an important function in drug delivery. Esterification of xylan via activation of carboxylic acid with N,N’-carbonyldiimidazole produces prodrugs for the controlled release of ibuprofen [28].

In the current paper, we investigated the anticancer efficacy of the PS 5-(4-hydroxyphenyl)-10,15,20-triphenylporphyrin (TPPOH). According to our research program on polysaccharide modifications with PS for PDT applications, we explored the newly synthesized core-shell hybrid SNPs based on xylan for the targeted delivery of TPPOH [29]. First, we showed a strong anticancer efficacy of TPPOH in human CRC cell lines, which has not been described before. Then, we demonstrated that vectorizing SNPs enhances TPPOH anticancer efficacy through increased uptake and ROS production due to optimal hydrophilicity and no aggregation of TPPOH. Subsequently, like other PDT studies, our results indicated that TPPOH achieves its anticancer efficacy through apoptosis. Because autophagy is frequently involved in PDT and plays a role in resistance to anticancer treatment [30,31], we established that inhibition of PDT-induced autophagy by pharmacological inhibitor markedly increases apoptosis. Finally, we highlighted that SNPs vectorization enhances TPPOH tumor cytotoxicity in a CRC xenograft mice model through better tumor-targeting due to the EPR effect. Together, the in vitro and in vivo results revealed the strong anticancer efficacy of TPPOH-X SNPs and showed strong potential for possible clinical use in further PDT therapies. 

## 2. Results

### 2.1. SNPs Vectorization Enhanced TPPOH-PDT Phototoxic Effects Mediated by ROS Production

**Figure 1:**
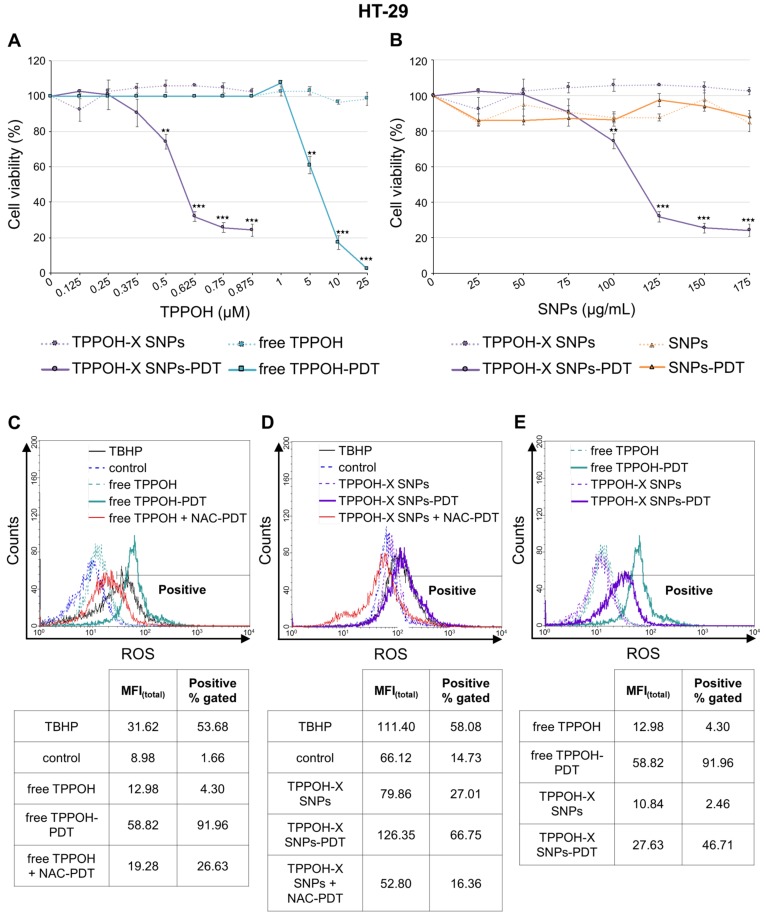
In vitro phototoxic effects of 5-(4-hydroxyphenyl)-10,15,20-triphenylporphyrin (TPPOH)-X silica nanoparticles (SNPs)- Photodynamic therapy (PDT) and reactive oxygen species (ROS) production. (**A**) HT-29 cells were treated or not treated with free TPPOH and TPPOH-X SNPs based on TPPOH concentration. Then, cells were exposed or were not exposed to PDT. Phototoxic effects were determined 48 h post-PDT using the MTT assay. Cell viability, expressed as a percentage of each condition, was compared to controls. IC_50_ values were calculated using 550.2 nM for TPPOH-X SNPs-PDT and around 6 μM for free TPPOH-PDT. (**B**) HT-29 cells were treated or not treated with TPPOH-X SNPs and SNPs based on nanoparticles concentration. Then, cells were exposed or not exposed to PDT. Phototoxic effects were determined 48 h post-PDT using the MTT assay. Cell viability, expressed as a percentage of each condition, was compared to controls. (**C**) HT-29 cells were treated or not treated with free TPPOH or (**D**) TPPOH-X SNPs with or without NAC co-treatment and then photoactivated or not photoactivated. (**E**) Comparison of free TPPOH and TPPOH-X SNPs on ROS generation in HT-29 cells. Intracellular ROS levels using DCFDA staining were measured 4 h post-PDT by flow cytometry. A greater right shift implied higher fluorescence intensity resulting from higher amounts of 2’,7’-dichlorofluorescein (DCF) formation and thus greater ROS generation. Data are shown as mean ± SEM (n = 3). ** *p* < 0.01 and *** *p* < 0.001.

To examine the phototoxicity of TPPOH-PDT in vitro, we treated three human CRC cell lines: HT-29, HCT116, and SW620 with free TPPOH or TPPOH-X SNPs. Then, cells were exposed or not to PDT with red irradiation and phototoxic effects were determined 48 h post-PDT, using 3-(4,5-dimethylthiazol-2-yl)-2,5-diphenyltetrazolium bromide (MTT) assay. Free TPPOH and TPPOH-X SNPs had no toxic effects on HT-29 cells when cells were kept in the dark (Figure 1A). When photoactivated, free TPPOH or TPPOH-X SNPs induced a strong decrease in cell viability in a dose-dependent manner (Figure 1A). However, TPPOH-X SNPs-PDT was more effective than free TPPOH-PDT. SNPs alone had no toxic effect with or without photoactivation regardless of the concentrations tested (Figure 1B). The same results were seen in HCT116 (Figure S1A,B) and SW620 (Figure S2A,B) cell lines. 

IC_50_ values were calculated in order to compare free TPPOH-PDT vs. TPPOH-X SNPs-PDT. We observed that TPPOH-X SNPs-PDT was much more effective than free TPPOH-PDT in HT-29 cells with 10.8-fold more cytotoxicity with IC_50_ values of 550.2 nM for TPPOH-X SNPs-PDT and around 6 μM for free TPPOH-PDT [29]. Similar results were observed in HCT116 (40.5-fold) and SW620 (39.5-fold) cell lines with respective IC_50_ values of 72.6 and 75.4 nM for TPPOH-X SNPs-PDT and around 3 μM for free TPPOH-PDT. HT-29 cells appeared to be the most resistant as IC_50_ values for free TPPOH-PDT and TPPOH-X SNPs-PDT were higher than those found for HCT116 and SW620 cell lines (2- and 7-fold respectively). For the following experiments, compounds were used at IC_50_ values except for during uptake and localization experiments. 

PDT-induced cell death generally occurs through generation of intracellular ROS. Therefore, we measured intracellular ROS levels using 2’,7’-dichlorofluorescein diacetate (DCFDA) staining 4 h post-PDT. Flow cytometry analyses indicated that exposure of cells to free TPPOH enhanced intracellular ROS levels only after photoactivation (Figure 1C). The median fluorescence intensity of 2’,7’-dichlorofluorescein (DCF) after photoactivated free TPPOH treatment was increased compared to free TPPOH and control and was decreased after pretreatment with the ROS scavenger: NAC. After SNPs vectorization, TPPOH-X SNPs also enhanced intracellular ROS levels only after photoactivation. Pretreatment with NAC decreased further the median fluorescence intensity of DCF (Figure 1D). Free TPPOH-PDT was more effective on ROS generation than TPPOH-X SNPs (Figure 1E). In fact, it is well-known complexation of PS to NPs often leads to a decrease of ROS generation through PS quenching [32,33]. TBHP was used as a positive control. The same results were observed in HCT116 (Figure S1C–E) and SW620 (Figure S2C–E) cell lines.

### 2.2. SNPs Vectorization Increased TPPOH Accumulation in Lysosomes

**Figure 2:**
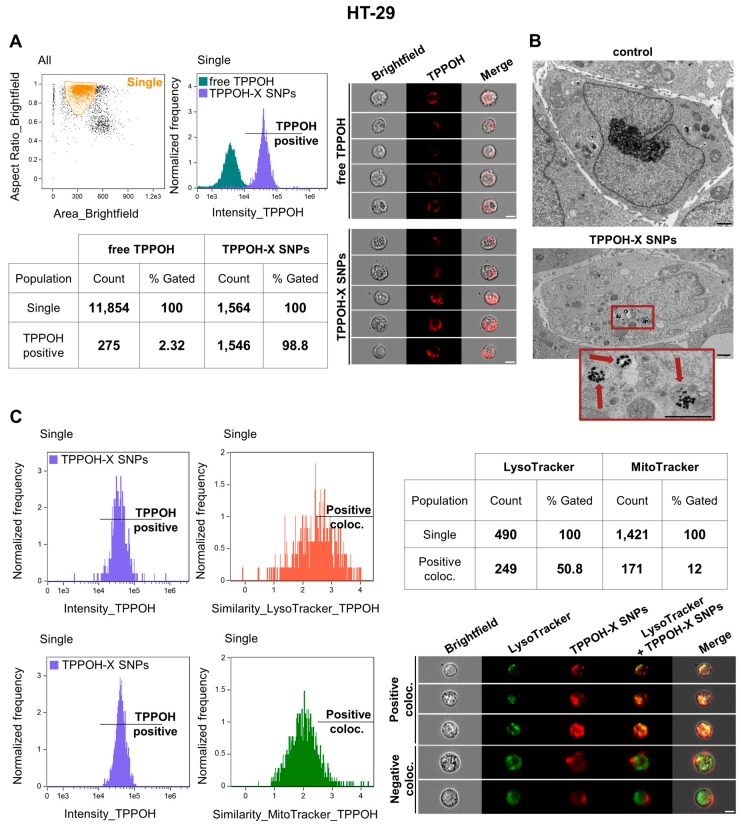
Cell uptake of TPPOH-X SNPs by HT-29 cells. (**A**) HT-29 cells were treated with free TPPOH and TPPOH-X SNPs at 1 μM and cell uptake of these compounds was studied 24 h post-treatment by AMNIS^®^ imaging flow cytometry. The first graph highlights the size/structure of HT-29 cells. After selection of the cell population, TPPOH intensity in HT-29 cells was shown in the second graph and in representative images. The table summarizes the amount of positive TPPOH cells relative to all cells compared to free TPPOH and TPPOH-X SNPs treatments. White scale bar = 10 μm. (**B**) Representative TEM images of HT-29 cells treated or not treated with TPPOH-X SNPs 24 h post-treatment are shown. Red arrows indicate intracellular nanoparticles. Black scale bar = 1 μm. (**C**) HT-29 cells were co-treated with TPPOH-X SNPs and LysoTracker or MitoTracker and co-localization was studied 24 h post-treatment by using AMNIS^®^ imaging flow cytometry analysis. The first graph shows TPPOH intensity in HT-29 cells and the second graph shows similarity of TPPOH positive cells compared to LysoTracker or MitoTracker. The table summarizes the amount of TPPOH positive cells co-localized with LysoTracker or MitoTracker. Representative images of co-localization of TPPOH-X SNPs and LysoTracker in HT-29 cells are shown. White scale bar = 10 μm. Data are shown as three independent experiments.

To explore the large difference in IC_50_ values between TPPOH-X SNPs-PDT and free TPPOH-PDT, we studied the uptake of these compounds through a kinetics study in human CRC cell lines by using AMNIS^®^ imaging flow cytometry analysis. The results demonstrated that, used at the same concentration (1 μM), TPPOH-X SNPs uptake was much higher in HT-29 cells than that of free TPPOH with 98.8% vs. 2.32%, respectively, 24 h post-treatment (Figure 2A). The same results were observed at 2, 6, and 12 h post-treatment (data not shown). TPPOH fluorescence (red) was clearly observed in cell cytoplasm, indicating cellular internalization. The same results were demonstrated in HCT116 (Figure S3A) and SW620 (Figure S4A) cell lines for TPPOH-X SNPs and free TPPOH with 99.9% vs. 0.53% and 99.8% vs. 0.9%, respectively. 

To visualize uptake on a single cell level, TEM analysis was used to evaluate the localization of TPPOH-X SNPs (Figure 2B). Images showed TPPOH-X SNPs uptake without cellular morphological changes. TPPOH-X SNPs seemed to be internalized by lysosomes probably by endocytosis and/or diffusion through cell membranes. TPPOH-X SNPs internalization seemed to be the same for HCT116 (Figure S3B) and SW620 (Figure S4B) cell lines. 

Then cells were co-treated with TPPOH-X SNPs and LysoTracker or MitoTracker. Results demonstrated that TPPOH-X SNPs co-localized preferentially with lysosomes (50.8%) compared to mitochondria (12%) in HT-29 cells (Figure 2C). TPPOH-X SNPs co-localized similarly in HCT116 (Figure S3C) and SW620 (Figure S4C) cell lines with lysosomes at 49.7% and 59.3% compared to mitochondria at 15% and 5.4%, respectively. TPPOH-X SNPs fluorescence co-localized preferably with LysoTracker fluorescence, as indicated by yellow fluorescence. TPPOH-X SNPs lysosomal localization was confirmed by confocal microscopy in HT-29 (Figure S5A), HCT116 (Figure S6A) and SW620 (Figure S7A) cell lines. In addition, confocal microscopy analysis revealed no co-localization between TPPOH-X SNPs and mitochondria in HT-29 (Figure S5B), HCT116 (Figure S6B), and SW620 (Figure S7B) cell lines. These data showed that SNPs vectorization enhanced cellular uptake and lysosome internalization compared to free TPPOH.

### 2.3. TPPOH-X SNPs-PDT Induced Apoptosis 

**Figure 3:**
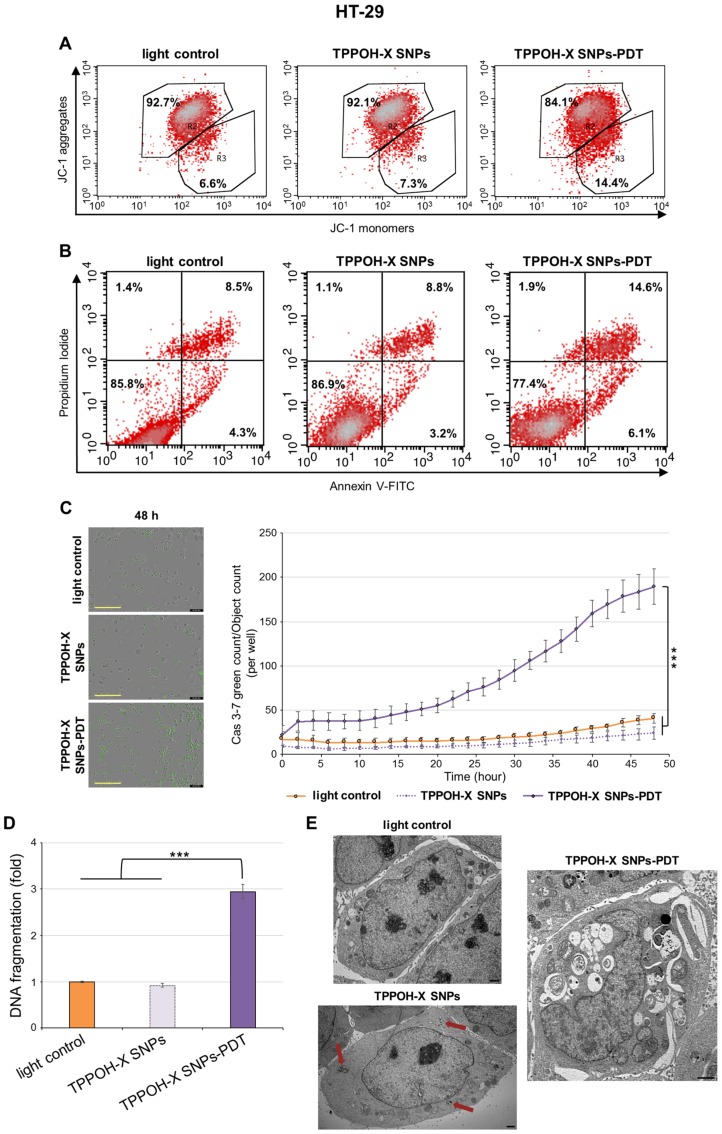
Effects of TPPOH-X SNPs-PDT on HT-29 cell line apoptosis. (**A**) HT-29 cells were treated or not treated with TPPOH-X SNPs and then photoactivated or not photoactivated. The mitochondrial membrane potential was analyzed by flow cytometry with JC-1 at 48 h post-PDT. R2 represents the aggregate ratio and R3 represents the monomer ratio. (**B**) HT-29 cells were also stained, 48 h post-PDT, with Annexin V-FITC and PI, and apoptosis was analyzed by flow cytometry. The upper right quadrant represents the percentage of late apoptosis, and the lower right quadrant represents early apoptosis. (**C**) Caspase-3/7 activity, with the same conditions in HT-29 cells, was evaluated every 2 h during 48 h post-PDT by IncuCyte imaging live cell analysis and green count/cell count/well are shown. Representative images at 48 h post-PDT are shown. Yellow scale bar = 400 μm. (**D**) DNA fragmentation in HT-29 cells 48 h post-PDT was quantified from cytosol extracts by ELISA. Results are reported as n-fold compared to light control. (**E**) Representative TEM images of HT-29 cells treated or not treated with TPPOH-X SNPs and photoactivated or not 48 h post-PDT were shown. Red arrows indicate intracellular nanoparticles. Black scale bar = 1 μm. Data are shown as mean ± SEM (n = 3). *** *p* < 0.001.

To determine the mechanism of the decrease in cell viability induced by TPPOH-X SNPs-PDT on human CRC cell lines, mitochondrial membrane potential was evaluated by flow cytometry using the cationic dye JC-1. In control cells, JC-1 forms J-aggregates in mitochondria. In apoptotic cells, JC-1 accumulates in the cytoplasm as monomers due to collapse of the mitochondrial membrane potential. Mitochondria predominantly exhibited accumulation of J-aggregates in HT-29 cells after light control or TPPOH-X SNPs treatment (Figure 3A). TPPOH-X SNPs-PDT disrupted mitochondrial membrane potential as revealed by an increase in monomer rates: 14.4% compared to 6.6% and 7.3% for light control and TPPOH-X SNPs, respectively. The same increase in monomer rates were demonstrated in HCT116 cells (Figure S8A) with 42.6% compared to 6.3% and 7.2% and SW620 cells (Figure S9A) with 22.7% compared to 0.8% and 1.1% for TPPOH-X SNPs-PDT relative to light control and TPPOH-X SNPs treatments, respectively. 

Consequently, the rate of apoptosis induced by TPPOH-X SNPs-PDT was determined by dual staining with Annexin V-FITC and PI by flow cytometry. In HT-29 cells, light control and TPPOH-X SNPs treated cells were mostly viable, whereas the rate of early and late apoptosis simultaneously increased with TPPOH-X SNPs-PDT to 20.7% compared to 12.8% and 12% for light control and TPPOH-X SNPs, respectively (Figure 3B). HCT116 and SW620 cells were more sensitive than HT-29 cells. TPPOH-X SNPs-PDT increased the rate of early and late apoptosis to 33.8% compared to 15% and 14% in HCT116 cells (Figure S8B) and to 30.4% compared to 12.1% and 15.1% in SW620 cells (Figure S9B) for TPPOH-X SNPs-PDT relative to light control and TPPOH-X SNPs, respectively. 

To further confirm that TPPOH-X SNPs-PDT induced apoptosis, we characterized the effects of TPPOH-X SNPs-PDT on activity of the key apoptosis executioner caspase-3/7 by IncuCyte imaging live cell analysis. HT-29 cells submitted to TPPOH-X SNPs-PDT showed a significant increase in caspase-3/7 activity in a time-dependent manner compared to light control or TPPOH-X SNPs (Figure 3C). At 48h, TPPOH-X SNPs-PDT induced a significant increase in caspase-3/7 activity by 4.6-fold ± 0.2-fold compared to light control. The same results were observed in HCT116 (Figure S8C) and SW620 (Figure S9C) cell lines with a significant increase in caspase-3/7 activity by 4.1-fold ± 0.1-fold and by 4.4-fold ± 0.1-fold, respectively, compared to light control. 

Furthermore, to study the nuclear changes in apoptosis caused by TPPOH-X SNPs-PDT, DNA fragmentation was evaluated by ELISA. TPPOH-X SNPs-PDT treatment induced a significant increase in DNA fragmentation by 2.9-fold ± 0.2-fold compared to light control or TPPOH-X SNPs in HT-29 cells (Figure 3D). The same results were seen in HCT116 (Figure S8D) and SW620 (Figure S9D) cell lines with a significant increase in DNA fragmentation by 2.5-fold ± 0.1-fold and by 2.4-fold ± 0.1-fold, respectively, compared to light control. 

TEM was also used to assess the apoptotic effects of TPPOH-X SNPs-PDT. The results showed that HT-29 (Figure 3E), HCT116 (Figure S8E) and SW620 cells (Figure S9E) treated with light control and TPPOH-X SNPs exhibited normal morphology with intact cell structures and undamaged nuclei. However, human CRC cell lines submitted to TPPOH-X SNPs-PDT showed a complete breakdown of intracellular structures. These cells exhibited morphological features such as cell membrane shrinkage, nuclear condensation and formation of phagocytic vesicles, or apoptotic bodies, which are hallmarks of apoptosis. These results demonstrated that TPPOH-X SNPs-PDT induced death of human CRC cell lines through apoptosis.

### 2.4. Autophagy Inhibition Enhanced TPPOH-X SNPs-PDT-Induced Apoptosis

For all Western blot figures, please include densitometry readings/intensity ratio of each band; please include the whole blot showing all the bands with all molecular weight markers on the Western area in the Supplementary Materials section.

**Figure 4:**
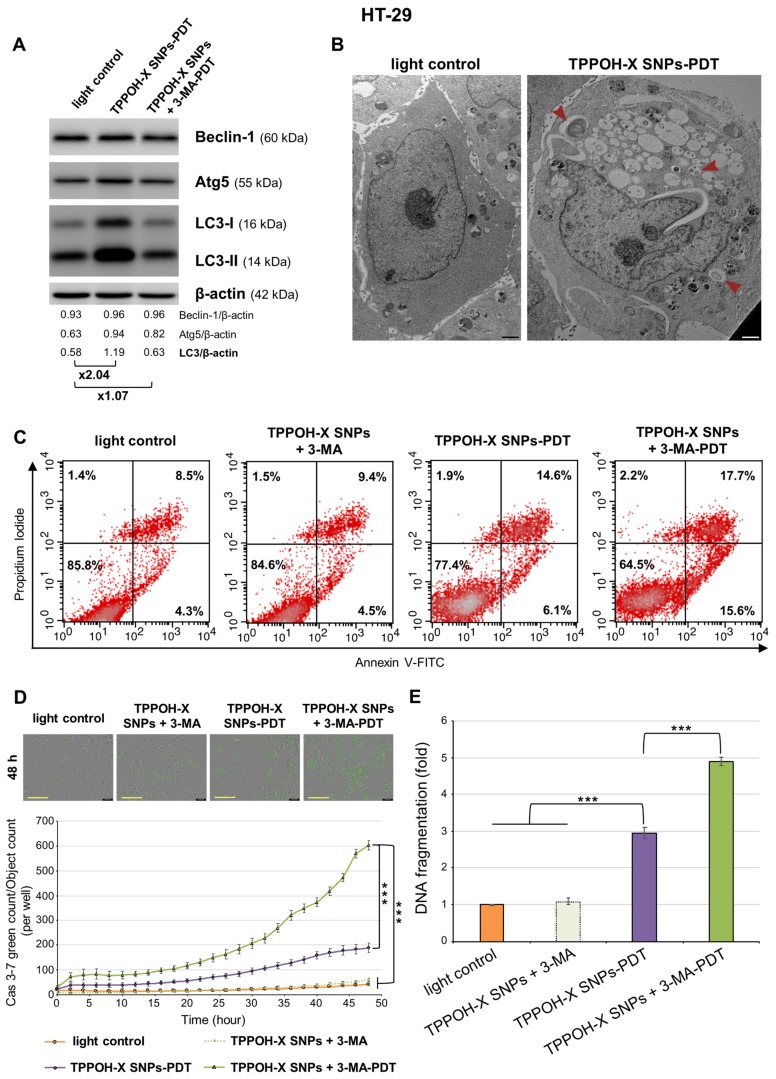
Effects of autophagy inhibition on HT-29 apoptosis. (**A**) HT-29 cells were treated or not with TPPOH-X SNPs in the presence or absence of 3-MA for 24 h. Expression of autophagy-related proteins was analyzed by Western blotting 48 h post-PDT. β-actin was used as a loading control. Representative images were shown. (**B**) Representative TEM images of HT-29 cells treated or not with TPPOH-X SNPs 48 h post-PDT protocol are shown. Red arrowheads indicate autophagosomes in the treated cells. Scale bar = 1 μm. (**C**) HT-29 cells were treated or not with TPPOH-X SNPs with or without 3-MA co-treatment and then were photoactivated or not photoactivated. At 48 h post-PDT, cells were stained with Annexin V-FITC and PI, and apoptosis was analyzed by flow cytometry. Upper right quadrant represents the percentage of late apoptosis, and the lower right quadrant represents early apoptosis. (**D**) With the same conditions of treatment, caspase-3/7 activity was evaluated each 2 h during 48 h post-PDT protocol by IncuCyte imaging live cell analysis and green count/cell count/well were shown. Representative images at 48 h post-PDT protocol were shown. Yellow scale bar = 400 μm. (**E**) With the same conditions of treatment, DNA fragmentation was quantified from cytosol extracts with ELISA. Results were reported as n-fold compared to light control. Data are shown as mean ± SEM (*n* = 3). *** *p* < 0.001.

Because autophagy is often involved during PDT-treatments, we studied TPPOH-X SNPs-PDT effects on autophagy. Western blotting was performed on autophagy-related proteins, Beclin-1, and Atg5, two key regulators of autophagy and light chain 3 (LC3) forms which are involved in autophagosome formation. The results demonstrated that HT-29 cells expressed a slight increase in Beclin-1 and Atg5 protein levels and induced a higher conversion of LC3-I into LC3-II after TPPOH-X SNPs-PDT compared to light control, resulting in autophagy activation (Figure 4A). Then we used a pharmacological inhibitor of autophagy as co-treatment: 3-MA, which can block the early steps of autophagy. Cells treated with TPPOH-X SNPs + 3-MA-PDT expressed lower levels of autophagy-related proteins compared to cells exposed to TPPOH-X SNPs-PDT without co-treatment with 3-MA. Similar results were obtained in HCT116 (Figure S10A) and SW620 cells (Figure S11A). 

Next, to confirm the induction of autophagy, cells were examined by TEM. Light control cells had an integrated cell nucleus and discrete organelles. However, HT-29 (Figure 4B), HCT116 (Figure S10B), and SW620 cells (Figure S11B) exposed to TPPOH-X SNPs-PDT were seriously damaged with clear cytoplasm vacuolization, with many membrane-bound vesicles containing organelles, cellular fragments, and double-membrane autophagosomes. 

To determine whether this autophagy induction is a key mediator in resistance to TPPOH-X SNPs-PDT in human CRC cells, we examined whether inhibition of autophagy by 3-MA enhanced TPPOH-X SNPs-PDT-induced apoptosis. First, effects of co-treatment with 3-MA on the rate of apoptosis were evaluated by dual staining with Annexin V-FITC and PI by flow cytometry. In HT-29 cells, TPPOH-X SNPs + 3-MA-PDT increased the rate of early and late apoptosis simultaneously compared to TPPOH-X SNPs-PDT by 33.3% vs. 20.7%, respectively. Moreover, HT-29 cells treated with TPPOH-X SNPs + 3-MA without irradiation were mostly viable, as were light control cells with 84.6% and 85.8% live cells, respectively (Figure 4C). The same results were obtained for HCT116 (Figure S10C) and SW620 cells (Figure S11C) with 52.1% vs. 33.8% and 37.8% vs. 30.4% increases for the rate of early and late apoptosis simultaneously after TPPOH-X SNPs + 3-MA-PDT and TPPOH-X SNPs-PDT, respectively. Similarly to HT-29 cells, HCT116 and SW620 cells co-treated with 3-MA were mostly viable with 83.4% and 83.7% increases compared to 82.7% and 86.4% for light control cells, respectively. 

In addition to these results, in HT-29 cells, co-treatment with 3-MA with TPPOH-X SNPs-PDT induced a significant increase in caspase-3/7 activity in a time-dependent manner (Figure 4D) and enhanced DNA fragmentation (Figure 4E) compared to TPPOH-X SNPs-PDT. Furthermore, co-treatment by 3-MA without PDT neither increased caspase-3/7 activity nor DNA fragmentation. The same results were found in HCT116 (Figure S10D,E) and SW620 (Figure S11D,E) cell lines. These data demonstrated in our study that autophagy acts as a resistance pathway of apoptosis.

### 2.5. SNPs Vectorization and Autophagy Inhibition Enhanced TPPOH-PDT Effects on Suppressing CRC Tumor Growth In Vivo

**Figure 5:**
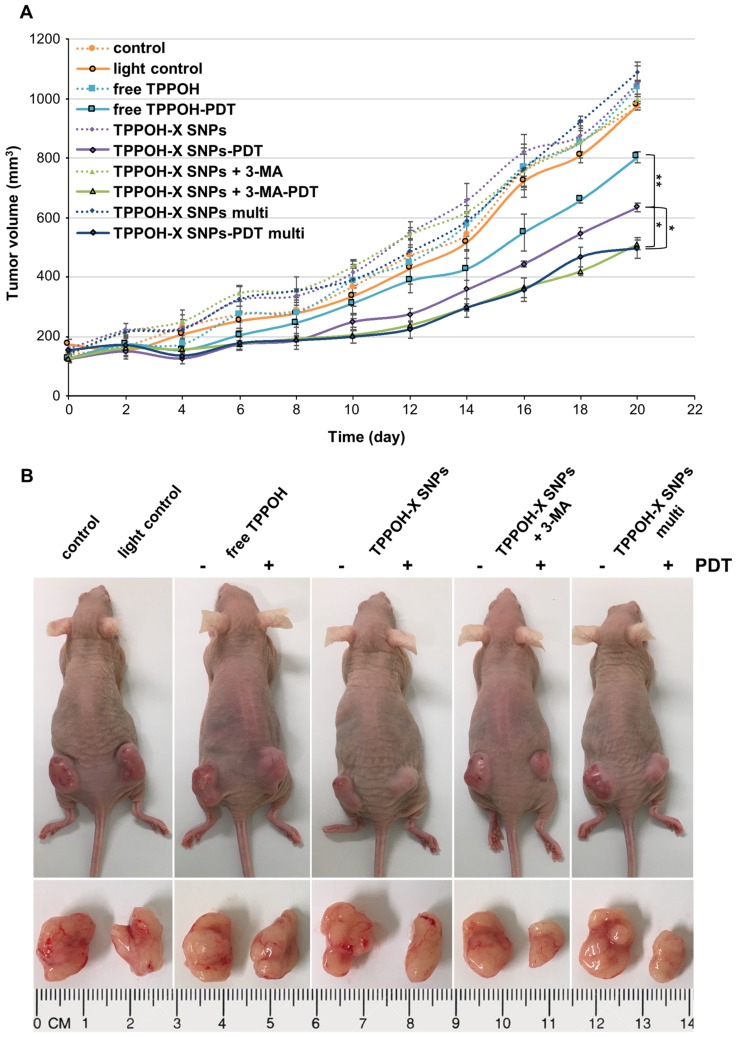
In vivo phototoxic effects on tumor growth. (**A**) Tumor growth curves of different groups over the treatment period until mouse sacrifice. (**B**) Representative images of HT-29 tumor-bearing nude mice and ex-vivo tumors after the mice being sacrificed on day 20. Data are shown as mean ± SEM (*n* = 5). * *p* < 0.05 and ** *p* < 0.01.

To test TPPOH-PDT phototoxic effects on tumor growth, we used a xenograft CRC tumor model. HT-29 cells, the most resistant cell line in our study, were injected subcutaneously into both flanks of Balb/c nude mice. When tumor volume reached 100–150 mm^3^, treatments were conducted by intravenous injection, at 1/100e LD_50_ for all TPPOH groups. After 24 h incubation, one tumor from each mouse was irradiated by laser. For the TPPOH-X SNPs multi group, the same protocol was conducted every 5 days. Tumor growth was recorded every 2 days during a period of 20 days (Figure 5A). In the control group, tumors exhibited rapid growth after seeding and no significant difference in tumor volume between light and non-light tumors was detected. This result indicated that light protocol did not suppressed tumorigenicity in vivo. However, TPPOH-PDT reduced tumor growth compared to TPPOH non-photoactivated treatments in all groups at the end point but with significant differences. TPPOH-PDT groups exhibited a slowing of tumor growth after approximately 2–4 days post-PDT. At the end point, free TPPOH-PDT induced a significant reduction in tumor growth by 22.5% ± 1.8% compared to free TPPOH non-PDT. TPPOH-X SNPs-PDT also induced a significant reduction of tumor growth by 37.7% ± 1.4% compared to non-photoactivated TPPOH-X SNPs. However, TPPOH-X SNPs-PDT was significantly more effective than free TPPOH-PDT. Moreover, tumor growth inhibition was significantly enhanced by 3-MA co-treatment. TPPOH-X SNPs + 3-MA-PDT significantly decreased tumor growth by 49% ± 1.7% vs. TPPOH-X SNPs + 3-MA non-PDT. However, TPPOH-X SNPs + 3-MA-PDT was significantly more efficient than TPPOH-X SNPs-PDT, with a significant reduction in tumor growth by 19.9% ± 0.3% compared to TPPOH-X SNPs-PDT. In addition, multi TPPOH-X SNPs-PDT were also efficient, with a significant reduction in tumor growth by 54.5% ± 3.2% vs. non-photoactivated multi TPPOH-X SNPs. Unfortunately, multi TPPOH-X SNPs-PDT did not induce the slowing of tumor growth but instead significantly increased tumor growth inhibition by 22.1% ± 1.3% compared to mono TPPOH-X SNPs treatment. Mouse body weight showed no significant difference between groups over the course of treatment (Figure S12A) indicating no systemic toxicity of free TPPOH or TPPOH-X SNPs. Mice were then sacrificed, and tumors were collected, recorded, and weighed. Tumor weights were consistent with tumor volumes. The tumor weight of TPPOH-PDT groups was in each case significantly decreased compared to the control or non-photoactivated TPPOH tumor groups (Figure S12B). These results were in agreement with the representative images showed for mouse and ex-vivo tumors from each group at the end point (Figure 5B).

### 2.6. TPPOH-X SNPs-PDT Induced Apoptosis In Vivo

**Figure 6:**
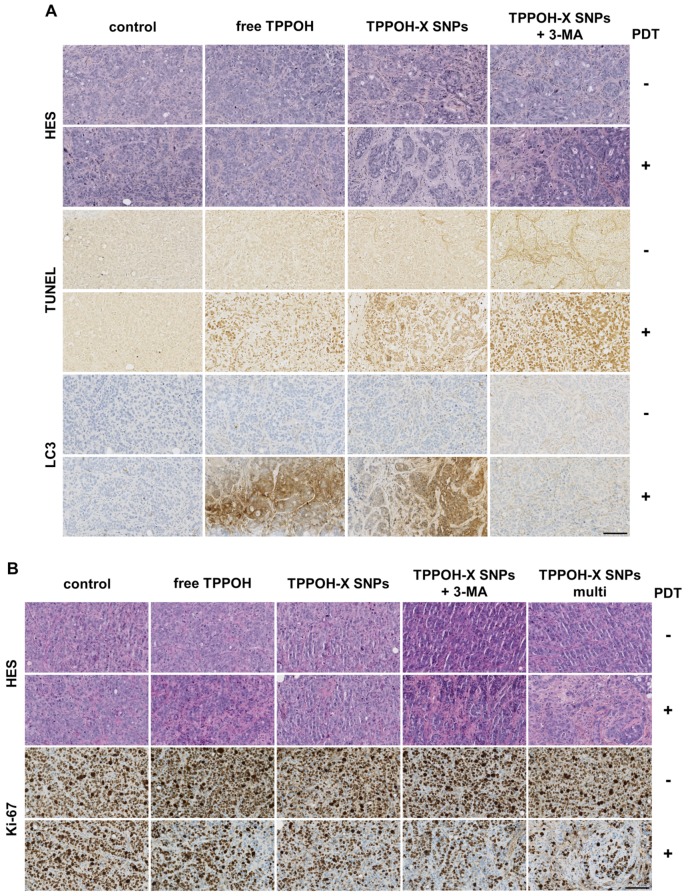
In vivo antitumor efficacy. (**A**) Tumors sections from treatment groups at 24 h post-PDT were stained with hematoxylin/eosin/saffron (HES), terminal deoxynucleotidyl transferase dUTP nick-end labeling (TUNEL) or LC3 staining. (**B**) Tumors sections from treatment groups after sacrifice were stained with HES and Ki-67. Representative images of each condition are shown. Black scale bar = 100 μm.

To estimate in vivo antitumor efficacy, histological analyses of tumors were performed 24 h post-PDT. One mouse from each group was sacrificed to determine the mechanism of cell death induced by our treatments. HES staining showed decreased cell density and increased tumor fibrosis after TPPOH-PDT, especially after TPPOH-X SNPs-PDT with or without 3-MA, which are evidence of tissue injury caused by oxidative damage (Figure 6A). Terminal deoxynucleotidyl transferase dUTP nick-end labeling (TUNEL)staining to assess the number of apoptotic cells, revealed apoptosis in tumors harvested from mice exposed to TPPOH-PDT. TPPOH-X SNPs-PDT with or without 3-MA induced the highest levels of apoptosis in tumors compared to free TPPOH-PDT which exhibited weak staining, indicating slight apoptosis. LC3 staining revealed 3-MA co-treatment efficacy, with a decrease in LC3 staining after TPPOH-X SNPs + 3-MA-PDT compared to TPPOH-PDT without 3-MA. According to precedent in vitro results, TPPOH-X SNPs + 3-MA-PDT induced the best antitumor response, revealed by strong levels of apoptosis in tumors compared to all TPPOH-PDT. Moreover, the comparison of PDT and non-PDT tumors, where almost all tumor cells were viable, validated the importance of laser irradiation as a trigger of apoptosis. 

At the end point, histological analyses of tumors were also performed. Consistent with the early HES staining, TPPOH-PDT induced a decrease in cell density and an increase in tumor fibrosis especially in TPPOH-X SNPs + 3-MA-PDT and multi TPPOH-X SNPs-PDT groups (Figure 6B). Ki-67 staining, a nuclear cell proliferation marker, showed that the number of cancer cells with a positively stained nucleus was markedly decreased after TPPOH-PDT compared to the control or non-PDT tumor groups. Consistent with the tumor growth results, SNPs vectorization of TPPOH showed a larger decrease in Ki-67 staining compared to the free TPPOH group. In addition, autophagy inhibition or multi TPPOH-X SNPs-PDT showed a marked decrease in Ki-67 staining compared to mono TPPOH-X SNPs-PDT. All these results demonstrating increased apoptosis and cell proliferation inhibition after TPPOH-PDT confirmed the in vivo antitumor efficacy of TPPOH-X SNPs.

### 2.7. SNPs Vectorization Enhanced Tumor-Targeting without Systemic Toxicity

**Figure 7:**
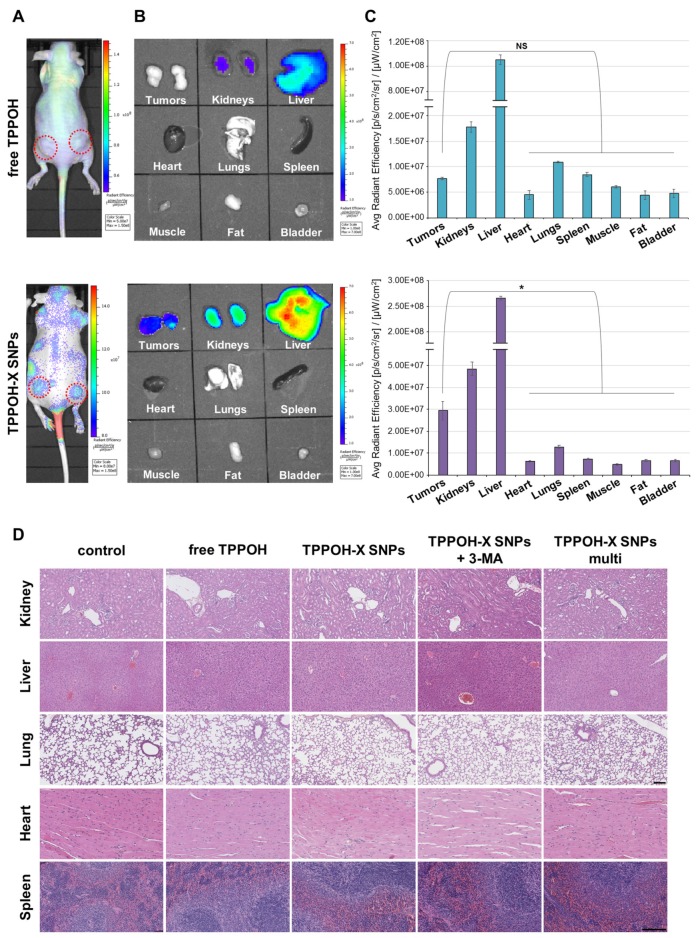
In vivo and ex vivo fluorescence imaging for biodistribution and safety evaluation. (**A**) In vivo fluorescence imaging of HT-29 tumor-bearing mice at 24 h post-intravenous injection of Cy5.5-labeled free TPPOH and TPPOH-X SNPs, at 1/100^e^ LD_50_ for each group. The red circles indicate tumor sites. (**B**) Ex-vivo fluorescence imaging of tumors and organs at 24 h post-injection. (**C**) ROI analysis of fluorescence intensity of tumors and organs at 24 h post-injection. (**D**) Representative images of histological analyses of major organ (kidney, liver, lung, heart and spleen) sections by HES staining. Black scale bar = 100 μm. Data are shown as mean ± SEM (*n* = 3). * *p* < 0.05 and NS: not significant.

In comparison with free drugs, tumor-specific accumulation through the EPR effect is a key characteristic of nano-scale drugs. Therefore, we explored the biodistribution of TPPOH-X SNPs in our HT-29 cell xenograft tumor model using the IVIS Lumina quantitative fluorescence imaging system. Cy5.5-labeled free TPPOH and TPPOH-X SNPs were administered intravenously at 1/100e LD_50_ for each group. As shown in Figure 7A, a strong TPPOH-X SNPs fluorescence signal was observed at tumor sites 24 h post injection. In contrast, free TPPOH displayed minimal accumulation at tumor sites and had a highly diffuse fluorescence pattern. To further verify the tumor-specific accumulation properties of TPPOH-X SNPs, ex-vivo fluorescence imaging of tumors and major organs was performed at 24 h post-injection (Figure 7B). In both cases, liver and kidney fluorescence intensities were higher than other organs or tumors. However, tumor fluorescence intensity of TPPOH-X SNPs was higher compared to other organs than that of free TPPOH. These observations were confirmed by quantitative ROI analysis (Figure 7C) which demonstrated that TPPOH-X SNPs displayed significantly better tumor-targeting than free TPPOH. 

To evaluate the potential systemic toxicity of our drug delivery system, sections of the major organs were stained with HES. In each case, including the TPPOH-X SNPs multi group, no damage was detected compared to the control group (Figure 7D). Pathological observations of SNPs treatments did not reveal significant differences, especially for hepatic inflammation or regeneration and renal impairment. These results highlighted the efficacy of TPPOH-X SNPs as having no toxic effects on the liver and kidneys, despite the high accumulation of SNPs on these organs at this dose.

## 3. Discussion

To increase the delivery of hydrophobic porphyrins to target sites, nanotechnology using nanocarriers appears to be the most promising strategy. NPs vectorization through encapsulation or attachment of PS not only enhances tumor-targeting through the EPR effect, but also increases PS hydrophilicity and tissue lifetime. Roy et al. entrapped the 2-devinyl-2-(1-hexyloxyethyl) pyropheophorbide (HPPH) into SNPs [34]. They reported efficient uptake, therefore significant cell death, after light irradiation compared to free drug in ovarian and cervical cancer cell lines. Secret et al. demonstrated similar results using anionic porphyrin-grafted porous silicon nanoparticles in breast cancer cells [35]. Other studies using chlorins as PS reported enhanced uptake using Chlorin e6 (Ce6) SNPs compared to Ce6 free carrier and also a stronger decrease in cell viability after Ce6 SNPs PDT compared to Ce6 alone in glioblastoma cancer cells [36] and breast cancer cells [37]. In addition, Brezániová et al. demonstrated that temoporfin SNPs enhanced uptake and cell death in breast cancer cells but also in MDA-MB-231 tumor bearing mice. They reported efficient antitumor responses using SNPs vectorization of temoporfin compared to free drug usage due to better tumor targeting through the EPR effect [38]. Furthermore, Simon et al. highlighted that protoporphyrin IX (PpIX) SNPs have better uptake and significantly enhanced cell death compared to free PpIX, which was confirmed by a strong fluorescence signal of ROS in HCT116 and HT-29 CRC cell lines. They reported that PpIX SNPs resulted in better tumor accumulation in HCT116 tumor bearing mice than the control alone, highlighting a greater selectivity for tumor tissues with SNPs vectorization [39]. Moreover, xylan could probably play an important role in controlled drug release. Sauraj et al. reported increased anticancer efficacy of xylan-stearic acid/5-fluorouracil NPs [40] and xylan-curcumin NPs [41] in HT-29 and HCT-15 CRC cell lines compared to free drugs. Xylan appears to be an efficient system for the delivery of hydrophobic anticancer drugs in cancer therapy. In our study, we compared the interest of TPPOH-X SNPs compared to free TPPOH. We demonstrated very significant phototoxic effects of TPPOH-X SNPs mediated by ROS generation post-PDT compared to free TPPOH in CRC cell lines. This improvement in anticancer activity shown by the decreased IC_50_ dose after SNPs vectorization was due to efficient cell uptake compared to free carrier TPPOH. In vivo, we improved antitumor efficacy using TPPOH-X SNPs-PDT compared to free TPPOH due to better tumor targeting through the EPR effect, as shown by increased accumulation of TPPOH-X SNPs in tumors compared to free TPPOH. These findings demonstrated a strong interest in SNPs vectorization for hydrophobic drug delivery in vitro and mainly in vivo with efficient tumor targeting. 

The major issues of inorganic SNPs are biosafety and toxicity. SNPs are one of the most biocompatible materials. However, diverse results have been reported about the safety of SNPs. The adverse effect depends on cell type and NPs size. Liu et al. demonstrated that 20 nm SNPs significantly induced apoptosis in a dose dependent manner from 100 μg/mL in human umbilical vein endothelial cells [42]. In contrast, Sergent et al. reported no cytotoxicity for 25 nm SNPs and limited cytotoxicity for 100 nm SNPs in HT-29 cells [43]. Cho et al. investigated the effect of the particle size on tissue distribution and tissue injury in vivo. They showed an accumulation in liver and spleen for 50 nm, 100 nm, and 200 nm SNPs, and observed a hepatic inflammatory response after injection of 100 and 200 nm SNPs. However, this effect was not reported for the smaller particles [44]. Moreover, Kumar et al. reported that 20 nm SNPs accumulated in all organs without signs of organ toxicity [45]. Although NPs size is important, the predominant issue is the dose injected. Chan et al. found no toxic effect in vivo after 150 nm SNPs intravenous injection up to 300 mg/kg [46]. Liu et al. demonstrated no side effects in vivo after up to 500 mg/kg of 110 nm SNPs intravenously. Repeated doses of 20, 40, and 80 mg/kg by continuous intravenous administration for 14 days have shown no toxicity. Nevertheless, for single dose toxicity, the LD_50_ was higher than 1 g/kg [47]. In our study, we did not detect any cytotoxicity for our 80 nm SNPs in HT-29 cells up to 175 μg/mL and in HCT116 and SW620 cell lines up to 35 μg/mL. We also explored the effect of 80 nm SNPs on apoptosis. Administration of 80 nm SNPs did not induce apoptosis in CRC cell lines and CRC cells exhibited normal morphology with intact cellular structures and undamaged nuclei on TEM (data not shown). In vivo, we did not observe any toxicity of mono or multi intravenous administration of SNPs at 270 mg/kg. SNPs accumulated especially in the liver and kidneys but with no signs of hepatic inflammation or renal impairment (data not shown). These results attest to the low toxicity of SNPs in vitro and in vivo when intravenous injection at single dose or repeated administrations.

The mode of cellular photodamage that occurs after PDT often involves death pathways such as necrosis or apoptosis. Necrosis is generally accompanied by a loss of membrane integrity and metabolic homeostasis due to uncontrolled and immediate cellular disintegration. This death pathway is associated with characteristic morphologic changes including cell swelling and membrane rupture. In general, necrosis may occur in cells when high fluence and PS concentrations are being applied [48]. Cell death described after PDT is usually through apoptosis. Apoptosis involves controlled cell destruction and packaging of cell components in apoptotic bodies, which can be phagocytized. Apoptosis is characterized morphologically by cell shrinkage and other distinctive changes such as nuclear chromatin condensation, fragmentation of the nucleus, and segregation of the cell into apoptotic bodies [49]. ROS generated either in mitochondria or in the cytoplasm have been shown to be a potent inducer of apoptosis. PDT-induced ROS-generation triggers mitochondrial pore-opening leading to caspase activation and thus induces apoptotic cell death [50,51]. Some authors have shown that tetraphenylporphyrin (TPP) derivatives triggered PDT-induced apoptosis. Costa et al. reported that 5,10,15,20-Tetra (quinolin-2-yl) porphyrin (2-TQP) decreased cell viability after PDT in HT-29 CRC cells [52]. Baldea et al. demonstrated the meso-5,10,15,20-tetrakis (4-hydroxyphenyl) porphyrin (THOPP) induced apoptosis through caspase activation after PDT [53]. Liao et al. reported TPP derivatives possessing piperidine groups induced cell death after PDT in the QBC-939 cholangiocarcinoma cell line and antitumor efficacy in QBC-939 tumor-bearing mice [54]. Roby et al. described TPP free and TPP-loaded PEG-PE micelles induced apoptosis in murine Lewis lung carcinoma [55]. Wu et al. highlighted apoptosis induction after PDT using POCL treatment composed in part of TPP in HeLa cervical cancer cells or HeLa tumor-bearing mice [56]. In our study, we demonstrated that TPPOH-X SNPs-PDT induced in vitro and in vivo cell death through the apoptosis pathway due to ROS generation in CRC cell lines. We have shown that TPPOH-X SNPs-PDT triggered mitochondrial pore-opening by the increase in JC-1 monomer rates. We highlighted apoptosis was due to caspase-3/7 involvement leading to DNA fragmentation. In addition, we demonstrated by TEM that CRC cells exhibited morphological features such as cell membrane shrinkage, nuclear condensation and formation of phagocytic vesicles or apoptotic bodies, hallmark events of apoptosis. We also reported that TPPOH-X SNPs induced apoptosis in our HT-29 tumor-bearing mice, as shown by the TUNEL assay. 

Numerous studies have indicated that autophagy is activated after PDT as a result of ROS generation. Autophagy is an essential physiological process that functions to maintain cell homeostasis by removing dysfunctional or impaired cellular components and organelles [57]. There are some controversies regarding autophagy functions: autophagy plays a critical role both in programmed cell death and in survival processes. Several reports have demonstrated that PDT-induced autophagy significantly improved cytoprotective effects. Xue et al. showed that Ce6-mediated PDT induced significant apoptosis and autophagy, as indicated by the increased expression of cleaved caspase-3 and enhanced conversion of LC3-I/II forms. Autophagy inhibition by the pharmacological inhibitor: 3-MA, markedly increased PDT-induced cell death in SW620 cells [58]. Xiong et al. demonstrated the same results with also an increased number of autophagic vacuoles using Photosan-mediated PDT in HCT116 and SW620 cells. Combined treatment with the autophagy inhibitor: chloroquine, aggravated apoptosis. This combined strategy also resulted in a greater killing effect in a xenograft model, in which tumor volume decreased faster in the combined group than in the group treated with PDT alone [59]. Wei et al. reported same data in vitro and in vivo using PpIX-mediated PDT and autophagy pharmacological inhibitors or Atg5 depletion in CRC cells [60]. However, autophagy can also act as a pro-death process. Some studies showed that applying an autophagy inhibitor significantly decreased cytotoxicity and apoptosis in osteosarcoma cells [61] or in breast cancer cells [62] treated with PDT. In our study, autophagy was involved after TPPOH-X SNPs-PDT in vitro as shown by the overexpression of autophagy-related proteins (Beclin-1, Atg5 and LC3) and the increased number of autophagic vacuoles. Autophagy is also enhanced in vivo as shown by the increased LC3 immunohistochemistry staining. Pharmacological autophagy inhibition by 3-MA markedly increased PDT-induced cell death in CRC cell lines. Moreover, in vivo autophagy inhibition induced a significant decrease in tumor volume compared to TPPOH-X SNPs-PDT without 3-MA co-treatment. Taken together, these findings suggest that PDT-stimulated autophagy acts as a PDT-resistance mechanism in our CRC model.

## 4. Materials and Methods 

### 4.1. Materials

DMEM medium, DMEM red-phenol-free medium, RPMI 1640 medium, RPMI 1640 red-phenol-free medium, fetal bovine serum (FBS), L-glutamine and penicillin-streptomycin were purchased from Gibco BRL (Cergy-Pontoise, France). 3-(4,5-dimethylthiazol-2-yl)-2,5-diphenyltetrazolium bromide (MTT), N-acetyl-L-cysteine (NAC), 3-methyladenine (3-MA), anti-β-actin antibody, 5,5′,6,6′-tetrachloro-1,1′,3,3′-tetraethylbenzimidazolocarbocyanine iodide (JC-1) and cell death detection enzyme-linked immunosorbent assay^PLUS^ (ELISA) were obtained from Sigma-Aldrich (Saint-Quentin-Fallavier, France). Beclin-1, Atg5 and LC3 antibodies were acquired from Cell Signaling Technology—Ozyme (Saint-Quentin-en-Yvelines). 2’,7’-dichlorofluorescein diacetate (DCFDA) cellular ROS detection assay kit and goat anti-rabbit IgG H&L horseradish peroxidase (HRP) secondary antibody were purchased from Abcam (Paris, France). LysoTracker, MitoTracker, rabbit anti-mouse IgG-IgM H&L HRP secondary antibody, TO-PRO-3, annexin V-FITC and propidium iodide (PI) were obtained from Invitrogen—Thermo Fisher Scientific (Villebon-sur-Yvette, France). Immobilon Western Chemiluminescent HRP Substrate was acquired from Merck (Lyon, France). Caspase-3/7 green reagent was purchased from Sartorius (Göttingen, Germany). 

### 4.2. Synthesis of Free TPPOH and TPPOH-X SNPs

The synthesis and characterization of free TPPOH and TPPOH-X SNPs were recently published by our research team [29]. SNPs were synthesized with an 80 nm average diameter following the modified Stöber method. Free TPPOH was synthesized and was conjugated with xylan via an esterification reaction forming TPPOH-X which was used in the surface modification of SNPs. The presence of glucuronic acid groups on xylan results in the formation of ionic bonds on the surface of SNPs which is made cationic by (3-aminopropyl) triethoxysilane (APTES). SNPs vectorization did not induced changes in the TPPOH spectrum (supplementary data [29]). Stock solutions of free TPPOH (5 mg/mL) and TPPOH-X SNPs (20 mg/mL) were prepared in ethanol.

### 4.3. Cell Culture

Human CRC cell lines (HT-29, HCT116 and SW620) were purchased from the American Type Culture Collection (ATCC—LGC Standards, Molsheim, France). Cells were grown in DMEM medium for HT-29 cells and RPMI 1640 medium for HCT116 and SW620 cells. Cells were supplemented with 10% FBS, 1% L-glutamine and 100 U/mL penicillin and 100 μg/mL streptomycin. Cultures were maintained in a humidified atmosphere containing 5% CO_2_ at 37 °C. For all experiments, cells were seeded at 2.1 × 10^4^, 1.2 × 10^4^ and 1.5 × 10^4^ cells/cm^2^ for HT-29, HCT116 and SW620 cells respectively and culture medium was replaced by red phenol-free appropriate culture medium before PDT. Stock solutions of free TPPOH and TPPOH-X SNPs were diluted in culture medium to obtain the appropriate final concentrations. The same amount of vehicle (percentage of ethanol did not exceed 0.6%) was added to control cells.

### 4.4. In Vitro Phototoxicity of TPPOH-PDT

Phototoxicity was determined using MTT assay. Cells were seeded in 96-well culture plates and grown for 24 h in appropriate culture medium prior to exposure or not to TPPOH or SNPs. After 24 h incubation, cells were irradiated or not with 630–660 nm CURElight lamp at 75 J/cm^2^ (PhotoCure ASA, Oslo, Norway). MTT assay were performed 48 h after irradiation and cell viability was expressed as a percentage of each treatment condition by normalizing to untreated cells.

### 4.5. Intracellular ROS Generation by TPPOH-PDT

ROS generation was quantified using a cellular reactive oxygen species detection assay which uses the cell permeant reagent DCFDA. Cells were seeded in 6-well culture plates and were grown for 24 h prior to exposure or not to TPPOH at respective IC_50_ values. After 24 h incubation, cells were stained with DCFDA for 30 min at 37 °C. After washing, cells were irradiated or not. To confirm ROS inhibition, cells were pretreated with ROS scavenger NAC 1 h before PDT at 10 mM. ROS generation was examined by flow cytometry 4 h post-PDT. Tert-Butyl Hydrogen Peroxide (TBHP) was used as a positive control at 250 μM.

### 4.6. TPPOH Cellular Uptake and Localization 

Cells were seeded in 6-well culture plates and were grown for 24 h prior to exposure to free TPPOH or TPPOH-X SNPs at the same concentration (1 μM TPPOH). After 24 h incubation, TPPOH fluorescence (excitation/emission: 405/650 nm) was determined by AMNIS^®^ imaging flow cytometry analysis and studied with IDEAS software (Merck). To determine TPPOH-X SNPs localizations, cells were seeded and treated as described above and co-treated at 37 °C with 75 nM LysoTracker during 2 h or 150 nM MitoTracker during 45 min. TPPOH-X SNPs localizations were determined by AMNIS^®^ imaging flow cytometry and studied with IDEAS software using TPPOH fluorescence (excitation/emission: 405/650 nm) with LysoTracker fluorescence (excitation/emission: 578/603 nm) or MitoTracker fluorescence (excitation/emission: 490/516 nm). The same protocol was conducted for confocal microscopy analysis and photos were taken with a confocal microscope (laser Zeiss LSM 510 Meta—×1000)

### 4.7. Transmission Electron Microscopy (TEM)

Cells were seeded in 6-well culture plates and were grown for 24 h prior to exposure or not to TPPOH-X SNPs treatment. After 24 h of incubation, cells were recovered for uptake experiment. For apoptosis and autophagy experiments, cells were irradiated or not. 48 h post-PDT protocol or immediately after 24 h of incubation for uptake experiment, cells were then incubated in 1% osmium tetroxide solution for 30 min at room temperature, dehydrated with increasing ethanol concentrations, and embedded in epon. Cells were polymerized over 48 h at 60 °C and ultrathin sections (80–100 nm) were prepared. Grids were stained with uranyl acetate and lead citrate and examined with TEM JEM-1011 (JEOL, Croissy-sur-Seine, France) operated at 80 KeV.

### 4.8. Autophagy Detection and Inhibition 

Cells were seeded in 25 cm^2^ tissue culture flasks and were grown for 24 h prior to exposure or not to TPPOH-X SNPs at the IC_50_ value. After 24 h incubation, cells were treated or not with the autophagy pharmacological inhibitor: 3-MA at 2 mM and were irradiated. 48 h post-PDT, cells were recovered and lysed in RIPA lysis buffer. Protein levels were determined using the Bradford method. Western blotting was performed on autophagy-related proteins, anti-Beclin-1 (1:1000), anti-Atg5 (1:1000) and anti-LC3 (1:1000). Anti-β-actin (1:5000) was used as a loading control. After incubation with the appropriate secondary antibodies, blots were developed using the Immobilon Western Chemiluminescent HRP Substrate and G:BOX system (Syngene, Cambridge, UK).

### 4.9. In Vitro Apoptosis by TPPOH-X SNPs 

Cells were seeded in 25 cm^2^ tissue culture flasks and were grown for 24 h prior to exposure or not to TPPOH-X SNPs at the IC_50_ value. After 24 h incubation, cells were treated or not with 3-MA and were irradiated. Cells were recovered 48 h post-PDT and divided in three groups. The first group was used to estimate mitochondrial membrane potential using JC-1. Cells were treated with JC-1 (1 μg/mL) for 30 min at 37 °C and then with TO-PRO-3 (1 μM) and mitochondrial membrane potential was immediately evaluated by flow cytometry. The second group of cells was used to determine apoptosis by dual staining with Annexin V-FITC and PI. Cells were treated with Annexin V-FITC and PI (1.5 μM) for 15 min at room temperature and cell viability was determined by flow cytometry. The last group was used to assess DNA fragmentation. Histone release from the nucleus during apoptosis was analyzed using the Cell Death Detection ELISA^PLUS^ as previously described [63]. Cytosol extracts were obtained according to the manufacturer’s protocol. DNA fragmentation was measured and results were reported as n-fold compared to control. 

Cells were seeded in 96-well culture plates and were grown for 24 h prior to exposure or not to TPPOH-X SNPs at the IC_50_ value. After 24 h incubation, cells were treated or not with 3-MA and were irradiated. Then, cells were treated with caspase-3/7 green reagent (5 μM) and were placed in the IncuCyte S3 live cell analysis system (Sartorius). Cells were imaged every 2 h with 4 images/well in phase contrast and green fluorescence modes using a ×10 objective to detect apoptotic cells. Apoptotic level was quantified by the IncuCyte software (Sartorius) as caspase-3/7 green count/cell count/well.

### 4.10. Heterotopic CRC Model

To establish a subcutaneous xenograft model of human CRC, human CRC HT-29 cells (5 × 10^6^ cells in 100 μL of 50/50 PBS-matrigel) were subcutaneously injected in each side of the dorsal region of four-week-old female Balb/c nude mice (≈20 g). At these sites, the tumors were easily accessible for treatment and assessment of response. We measured tumor dimensions every other day by a caliper and calculated the volume with the formula (V = 4π/3 × LW^2^/8, where L is tumor length and W is tumor width) [64].

### 4.11. In Vivo Antitumor Efficacy and Biosafety Evaluation of TPPOH-PDT 

To confirm antitumor efficacy, HT-29 tumor-bearing mice were established as described above and anticancer treatments were administered when the tumors were approximately 100–150 mm^3^. Mice were randomly divided into five groups (*n* = 6): control, free TPPOH, TPPOH-X SNPs, TPPOH-X SNPs + 3-MA and TPPOH-X SNPs multi. Mice were injected in the tail vein with 100 μL phosphate buffered saline (PBS) or 1/100^e^ lethal dose 50 (LD_50_) (Log LD_50_ = 0.435 log IC_50_ (mM) + 0.625) [65] for all TPPOH groups: free TPPOH (3.26 mg/kg), TPPOH-X SNPs with or without 3-MA and multi group (1.16 mg/kg TPPOH and 334 mg/kg SNPs). Mice from the TPPOH-X SNPs + 3-MA group received a 100 μL IP injection of 24 mg/kg 3-MA [66]. Then, 24 h post-injection, only one tumor per mouse was subjected to light irradiation to compare intra-individual irradiation effects. Consequently, 10 conditions were studied: each of the 5 groups was divided in 2 conditions (no irradiation: PDT—and red irradiation: PDT +). Irradiation was performed with a 660 nm red laser (Z-LASER, Freiburg, Germany). PDT was performed by 2 sequences of 5 min irradiation separated by 5 min (at 660 nm with 100 mW, for a 200 J/cm^2^ fluence as previously described [67]. For the TPPOH-X SNPs multi group, the same protocol was conducted every 5 days. At 24 h post-PDT, one mouse from each group except for the TPPOH-X SNPs multi group was sacrificed and tumors were harvested and fixed in 4% paraformaldehyde to prepare paraffin sections. Hematoxylin/eosin/saffron (HES) staining was used for histological analyses, while TUNEL assay and LC3 staining were performed to assess apoptosis and autophagy levels in the tumors, respectively. For other mice, tumor size and mouse body weight were recorded every 2 days. HT-29 tumor-bearing mice were sacrificed on day 20 after initial drug treatment and tumor weight was recorded. Tumors and major organs including kidneys, liver, lungs, heart and spleen were harvested and fixed in 4% paraformaldehyde to prepare paraffin sections. HES staining was used for histological analysis and Ki-67 staining was used to assess tumor cell proliferation and growth. HES staining was performed with Tissue Tek (Sakura, Alphen aan den Rijn, Netherlands), Ki-67 staining with BenchMark Ultra Ventana (Roche Diagnostics, Meylan, France), TUNEL assay with the cell death detection kit POD (Roche Diagnostics) and LC3 staining with LC3 antibody (1:200) and revealed with Acuity Advanced Biotin Free Polymer Detection System DAB (BioLegend, London, UK). All histological analyses were scanned under the NanoZoomer RS2 optical microscope scanner (Hamamatsu Photonics, Massy, France) and studied with NDPView software.

### 4.12. In Vivo Biodistribution of TPPOH-X SNPs 

To determine the biodistribution of TPPOH treatments, free TPPOH and TPPOH-X SNPs were labeled with Cyanine 5.5 (Lumiprobe, Hannover, Germany) to allow tracking because the TPPOH emission spectrum overlaps with that of blood. HT-29 tumor-bearing mice, established as described above, were randomly divided into two groups (*n* = 3) and intravenously injected with 1/100^e^ LD_50_ for both group: Cy5.5-free TPPOH (3.26 mg/kg) and Cy5.5-TPPOH-X SNPs (1.16 mg/kg TPPOH and 334 mg/kg SNPs). Then 24 h post-injection, the biodistribution was determined using IVIS Lumina quantitative fluorescence imaging system (PerkinElmer, Villepinte, France). Subsequently, the mice were sacrificed and ex vivo biodistribution images of the tumors and major organs were immediately taken. Relative signal intensity in tumors and organs was calculated, using Living Image software (PerkinElmer), as radiant efficiency ([photons/s/cm^2^/sr]/[μW/cm^2^]) per pixel of the region of interest (ROI), which was drawn around the respective organ.

### 4.13. Ethical Statement

Institutional review board approval was obtained from the Regional Animal Experimentation Ethics Committee (approval number: #16335-2018073009499570 v3). All animal experiments and experimental protocols were in accordance with the recommendations of the European Directive of 22 September 2010 (2010/63/EU) on the protection of animals used for scientific purposes. All efforts were made to reduce the number of animals used and to ensure their optimal conditions of well-being before, during, and after each experiment.

### 4.14. Statistical Analysis

All quantitative results are expressed as the mean ± standard error of the mean (SEM) of separate experiments. Statistical significance was evaluated by the two-tailed unpaired Student’s t-test and expressed as: * *p* < 0.05; ** *p* < 0.01 and *** *p* < 0.001. 

## 5. Conclusions

In this study, we evaluated for the first time the anticancer efficacy of the new TPPOH-X SNPs synthesized by our research team in human CRC cell lines and on HT-29 tumor-bearing mice. According to our hypothesis concerning the interest of SNPs vectorization, we demonstrated the strong anticancer efficacy of TPPOH in vitro and in vivo, and the additional benefit of vectorized SNPs. As shown by the strong antitumor efficacy on HT-29 tumor-bearing mice with both multi TPPOH-X SNPs-PDT or co-treatment with an autophagy inhibitor, our new TPPOH-X SNPs seem to be a promising PDT agent for further clinical protocols.

## Supplementary Materials

The following are available online at https://www.mdpi.com/2072-6694/11/10/1474/s1, Figure S1: In vitro phototoxic effects of TPPOH-X SNPs-PDT and ROS production in HCT116 cells, Figure S2: In vitro phototoxic effects of TPPOH-X SNPs-PDT and ROS production in SW620 cells, Figure S3: Cell uptake of TPPOH-X SNPs by HCT116 cells, Figure S4: Cell uptake of TPPOH-X SNPs by SW620 cells, Figure S5: TPPOH-X SNPs localization in HT-29 cells, Figure S6: TPPOH-X SNPs localization in HCT116 cells, Figure S7: TPPOH-X SNPs localization in SW620 cells, Figure S8: Effects of TPPOH-X SNPs-PDT on HCT116 cell line apoptosis, Figure S9: Effects of TPPOH-X SNPs-PDT on SW620 cell line apoptosis, Figure S10: Effects of autophagy inhibition on HCT116 apoptosis, Figure S11: Effects of autophagy inhibition on SW620 apoptosis, Figure S12: In vivo phototoxic effects on tumor growth.

## Abbreviations

3-MA: 3-methyladenine; APTES: (3-aminopropyl) triethoxysilane; Ce6: Chlorin e6; CRC: colorectal cancer; DCFDA: 2’,7’-dichlorofluorescein diacetate; ELISA: enzyme-linked immunosorbent assay; EPR effect: enhanced permeability and retention effect; FBS: fetal bovine serum; HES: hematoxylin/eosin/saffron; HRP: horseradish peroxidase; JC-1: 5,5′,6,6′-tetrachloro-1,1′,3,3′-tetraethylbenzimidazolocarbocyanine iodide; LC3: light chain 3; LD_50_: lethal dose 50; MTT: 3-(4,5-dimethylthiazol-2-yl)-2,5-diphenyltetrazolium bromide; NAC: N-acetyl-L-cysteine; NPs: nanoparticles; PBS: phosphate buffered saline; PDT: photodynamic therapy; PI: propidium iodide; Protoporphyrin IX: PpIX; PS: photosensitizer; ROI: region of interest; ROS: reactive oxygen species; SEM: standard error of the mean; SNPs: silica nanoparticles; TBHP: tert-butyl hydrogen peroxide; TEM: transmission electron microscopy; TPP: tetraphenylporphyrin; TPPOH-X: xylan-TPPOH conjugate; TPPOH: 5-(4-hydroxyphenyl)-10,15,20-triphenylporphyrin; TUNEL: terminal deoxynucleotidyl transferase dUTP nick-end labeling.
