# Supplementary material for: Photodynamic Therapy Activity of New Porphyrin-Xylan-Coated Silica Nanoparticles in Human Colorectal Cancer
Source: Cancers (Basel). 2019 Sep 30;11(10):1474. doi: 10.3390/cancers11101474 (PMC6826978; doi:10.3390/cancers11101474)
Supplement: Supplementary file 1 [file cancers-11-01474-s001.pdf]

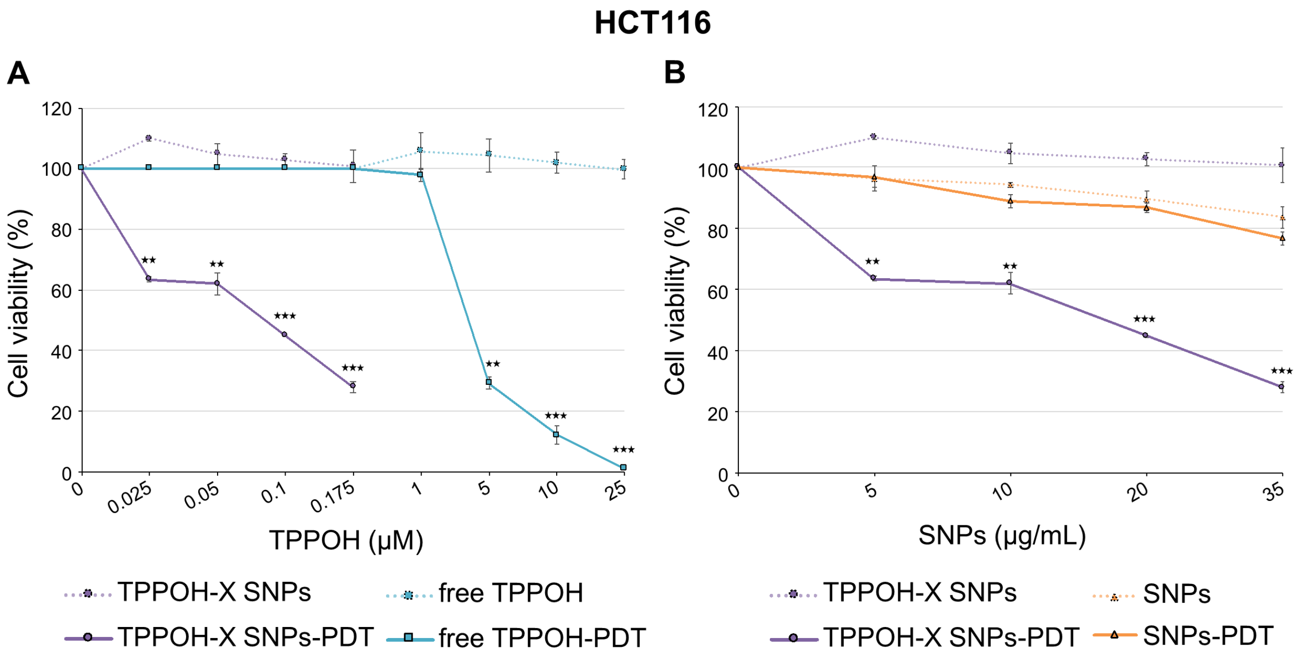

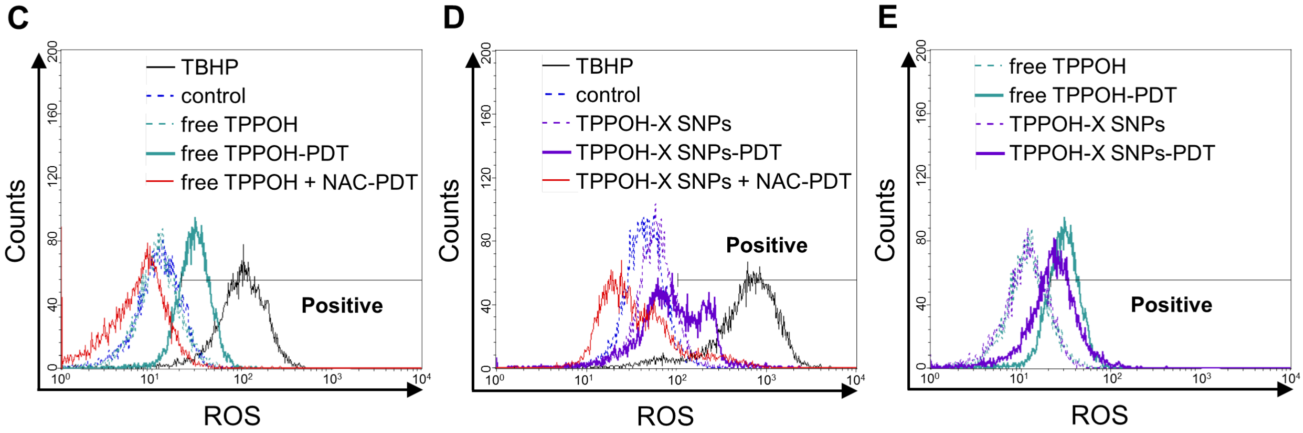

	$MFI_{(total)}$	Positive % gated
TBHP	103.66	98.19
control	12.63	13.80
free TPPOH	12.08	12.58
free TPPOH-PDT	29.69	81.08
free TPPOH + NAC-PDT	8.06	3.14

	$MFI_{(total)}$	Positive % gated
TBHP	661.17	95.11
control	45.32	3.51
TPPOH-X SNPs	59.35	9.67
TPPOH-X SNPs-PDT	87.38	41.66
TPPOH-X SNPs + NAC-PDT	29.96	11.03

	$MFI_{(total)}$	Positive % gated
free TPPOH	12.08	12.58
free TPPOH-PDT	29.69	81.08
TPPOH-X SNPs	11.97	11.60
TPPOH-X SNPs-PDT	23.29	57.35

Figure S1. *In vitro* phototoxic effects of TPPOH-X SNPs-PDT and ROS production. (**A**) HCT116 cells were treated or not with free TPPOH and TPPOH-X SNPs based on TPPOH concentration. Then, cells were exposed or not to PDT. Phototoxic effects were determined 48 h post-PDT using the MTT assay. Cell viability, expressed in percentage of each condition, was compared to controls. IC_{50} values were calculated of 72.6 nM for TPPOH-X SNPs-PDT and around 3 μM for free TPPOH-PDT. (**B**) HCT116 cells were treated or not with TPPOH-X SNPs and SNPs based on nanoparticles concentration. Then, cells were exposed or not to PDT. Phototoxic effects were determined 48 h post-PDT using the MTT assay. Cell viability, expressed in percentage of each condition, was compared to controls. (**C**) HCT116 cells were treated or not with free TPPOH or (**D**) TPPOH-X SNPs with or without NAC co-treatment and then photoactivated or not. (**E**) Comparison of free TPPOH and TPPOH-X SNPs on ROS generation in HCT116 cells. Intracellular ROS levels using DCFDA staining were measured 4 h post-PDT by flow cytometry. Greater right shift implied higher fluorescence intensity resulting from higher amounts of DCF formation and thus greater ROS generation. Data are shown as mean ± SEM (n = 3). **p < 0.01 and ***p < 0.001.

SW620

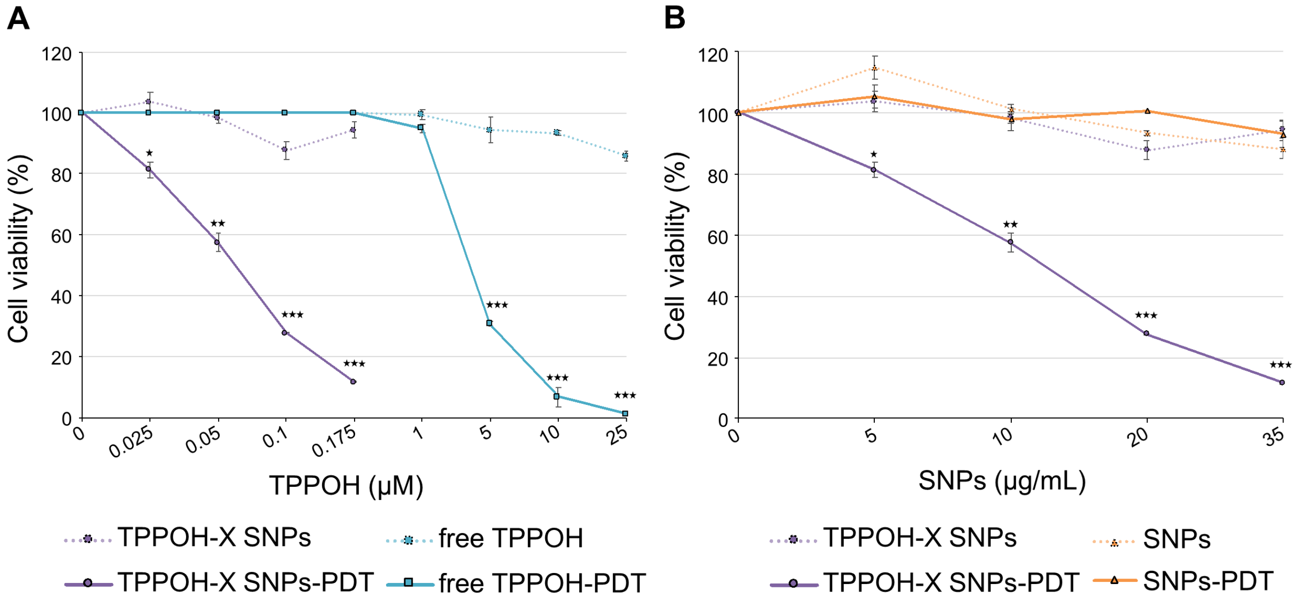

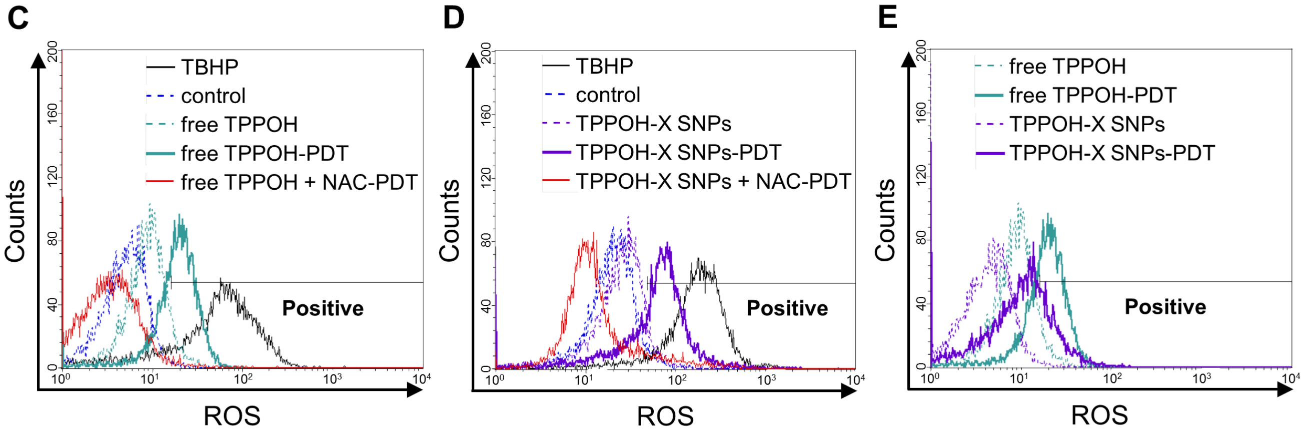

	**MFI$_{(total)}$**	**Positive % gated**
TBHP	56.74	82.48
control	5.28	0.72
free TPPOH	9.06	7.50
free TPPOH-PDT	19.46	66.76
free TPPOH + NAC-PDT	3.40	1.26

	**MFI$_{(total)}$**	**Positive % gated**
TBHP	182.69	93.97
control	21.10	3.42
TPPOH-X SNPs	26.18	7.31
TPPOH-X SNPs-PDT	68.54	71.21
TPPOH-X SNPs + NAC-PDT	11.04	7.99

	**MFI$_{(total)}$**	**Positive % gated**
free TPPOH	9.06	7.50
free TPPOH-PDT	19.46	66.76
TPPOH-X SNPs	4.49	0.54
TPPOH-X SNPs-PDT	10.84	25.69

Figure S2. *In vitro* phototoxic effects of TPPOH-X SNPs-PDT and ROS production. (**A**) SW620 cells were treated or not with free TPPOH and TPPOH-X SNPs based on TPPOH concentration. Then, cells were exposed or not to PDT. Phototoxic effects were determined 48 h post-PDT using the MTT assay. Cell viability, expressed in percentage of each condition, was compared to controls. IC_{50} values were calculated of 75.4 nM for TPPOH-X SNPs-PDT and around 3 μM for free TPPOH-PDT. (**B**) SW620 cells were treated or not with TPPOH-X SNPs and SNPs based on nanoparticles concentration. Then, cells were exposed or not to PDT. Phototoxic effects were determined 48 h post-PDT using the MTT assay. Cell viability, expressed in percentage of each condition, was compared to controls. (**C**) SW620 cells were treated or not with free TPPOH or (**D**) TPPOH-X SNPs with or without NAC co-treatment and then photoactivated or not. (**E**) Comparison of free TPPOH and TPPOH-X SNPs on ROS generation in SW620 cells. Intracellular ROS levels using DCFDA staining were measured 4 h post-PDT by flow cytometry. Greater right shift implied higher fluorescence intensity resulting from higher amounts of DCF formation and thus greater ROS generation. Data are shown as mean ± SEM (n = 3). **$p < 0.01$ and ***$p < 0.001$.

HCT116

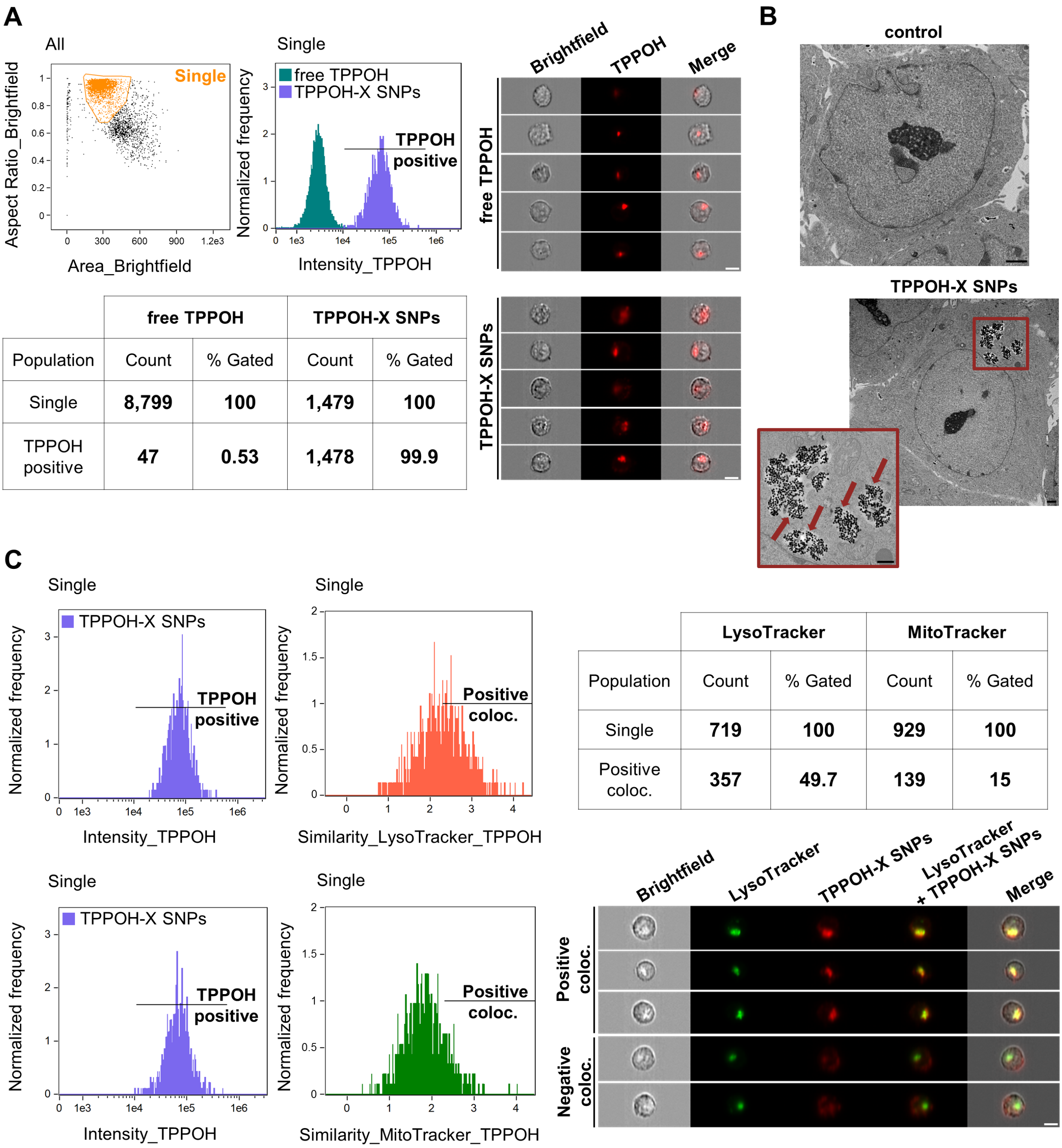

	free TPPOH		TPPOH-X SNPs	
Population	Count	% Gated	Count	% Gated
Single	**8,799**	**100**	**1,479**	**100**
TPPOH positive	**47**	**0.53**	**1,478**	**99.9**

	LysoTracker		MitoTracker	
Population	Count	% Gated	Count	% Gated
Single	**719**	**100**	**929**	**100**
Positive coloc.	**357**	**49.7**	**139**	**15**

Figure S3. Cell uptake of TPPOH-X SNPs by HCT116 cells. (**A**) HCT116 cells were treated with free TPPOH and TPPOH-X SNPs at 1 μM and cell uptake of these compounds was studied 24 h post-treatment by AMNIS imaging flow cytometry. The first graph highlights the size/structure of HCT116 cells. After selection of the cell population, TPPOH intensity in HCT116 cells was shown in the second graph and in representative images. The table summarizes the amount of positive TPPOH cells relative to all cells compared to free TPPOH and TPPOH-X SNPs treatments. White scale bar = 10 μm. (**B**) Representative TEM images of HCT116 cells treated or not with TPPOH-X SNPs 24 h post-treatment are shown. Red arrows indicate intracellular nanoparticles. Black scale bar = 1 μm. (**C**) HCT116 cells were co-treated with TPPOH-X SNPs and LysoTracker or MitoTracker and co-localization was studied 24 h post-treatment by AMNIS imaging flow cytometry analysis. The first graph shows TPPOH intensity in HCT116 cells and the second graph shows similarity of TPPOH positive cells compared to LysoTracker or MitoTracker. The table summarizes the amount of TPPOH positive cells co-localized with LysoTracker or MitoTracker. Representative images of co-localization of TPPOH-X SNPs and LysoTracker in HCT116 cells are shown. White scale bar = 10 μm. Data are shown as three independent experiments.

SW620

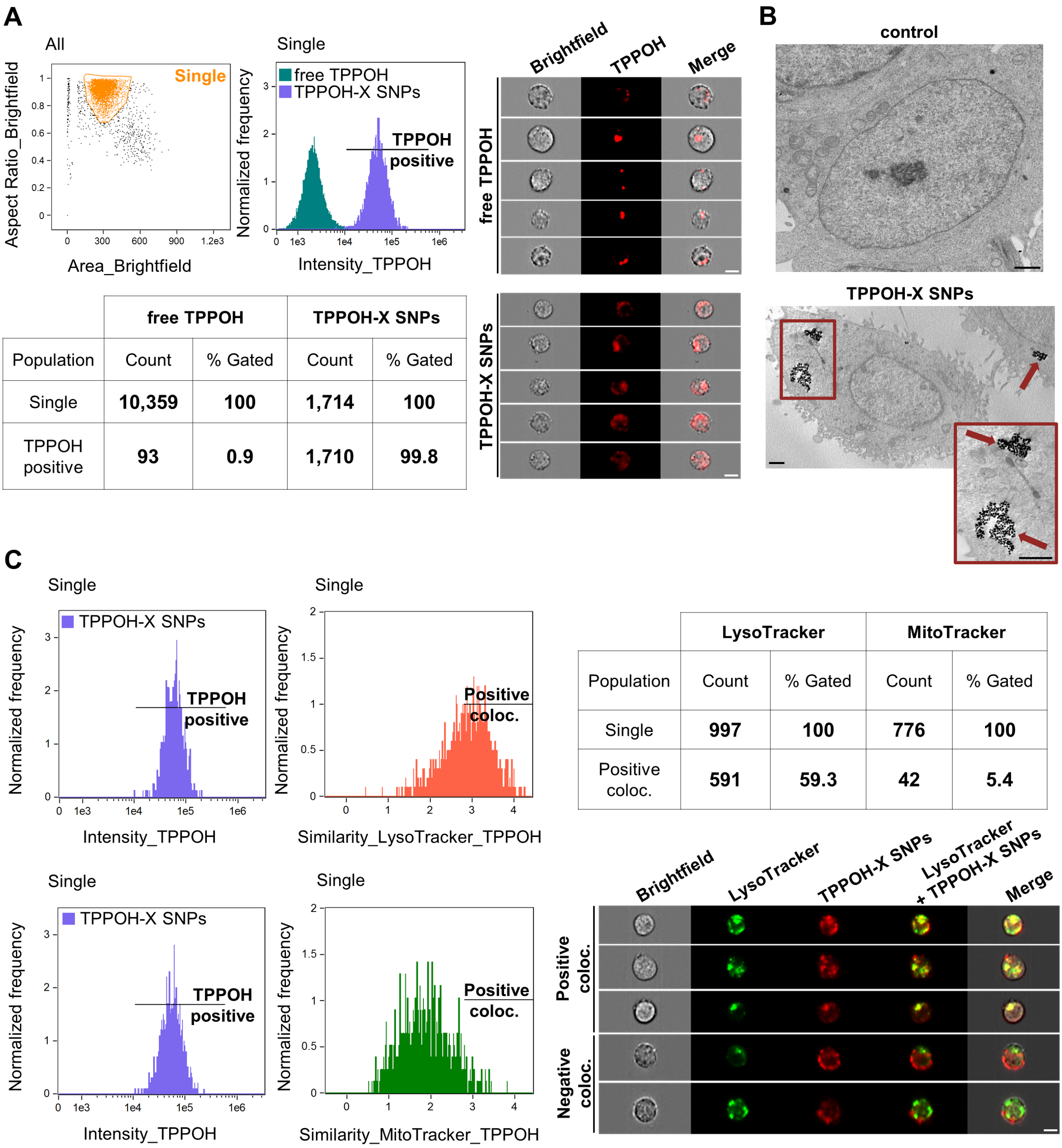
A
All
Single
Aspect Ratio_Brightfield
Area_Brightfield
Single
Normalized frequency
free TPPOH
TPPOH-X SNPs
TPPOH positive
Intensity_TPPOH
Brightfield
TPPOH
Merge
free TPPOH
TPPOH-X SNPs
free TPPOH | TPPOH-X SNPs
Population | Count | % Gated | Count | % Gated
Single | 10,359 | 100 | 1,714 | 100
TPPOH positive | 93 | 0.9 | 1,710 | 99.8
B
control
TPPOH-X SNPs
C
Single
TPPOH-X SNPs
TPPOH positive
Normalized frequency
Intensity_TPPOH
Single
Positive coloc.
Similarity_LysoTracker_TPPOH
LysoTracker | MitoTracker
Population | Count | % Gated | Count | % Gated
Single | 997 | 100 | 776 | 100
Positive coloc. | 591 | 59.3 | 42 | 5.4
Single
TPPOH-X SNPs
TPPOH positive
Intensity_TPPOH
Single
Positive coloc.
Similarity_MitoTracker_TPPOH
Brightfield
LysoTracker
TPPOH-X SNPs
LysoTracker + TPPOH-X SNPs
Merge
Positive coloc.
Negative coloc.

Figure S4. Cell uptake of TPPOH-X SNPs by SW620 cells. (**A**) SW620 cells were treated with free TPPOH and TPPOH-X SNPs at 1 μM and cell uptake of these compounds was studied 24 h post-treatment by AMNIS imaging flow cytometry. The first graph highlights the size/structure of SW620 cells. After selection of the cell population, TPPOH intensity in SW620 cells was shown in the second graph and in representative images. The table summarizes the amount of positive TPPOH cells relative to all cells compared to free TPPOH and TPPOH-X SNPs treatments. White scale bar = 10 μm. (**B**) Representative TEM images of SW620 cells treated or not with TPPOH-X SNPs 24 h post-treatment are shown. Red arrows indicate intracellular nanoparticles. Black scale bar = 1 μm. (**C**) SW620 cells were co-treated with TPPOH-X SNPs and LysoTracker or MitoTracker and co-localization was studied 24 h post-treatment by AMNIS imaging flow cytometry analysis. The first graph shows TPPOH intensity in SW620 cells and the second graph shows similarity of TPPOH positive cells compared to LysoTracker or MitoTracker. The table summarizes the amount of TPPOH positive cells co-localized with LysoTracker or MitoTracker. Representative images of co-localization of TPPOH-X SNPs and LysoTracker in SW620 cells are shown. White scale bar = 10 μm. Data are shown as three independent experiments.

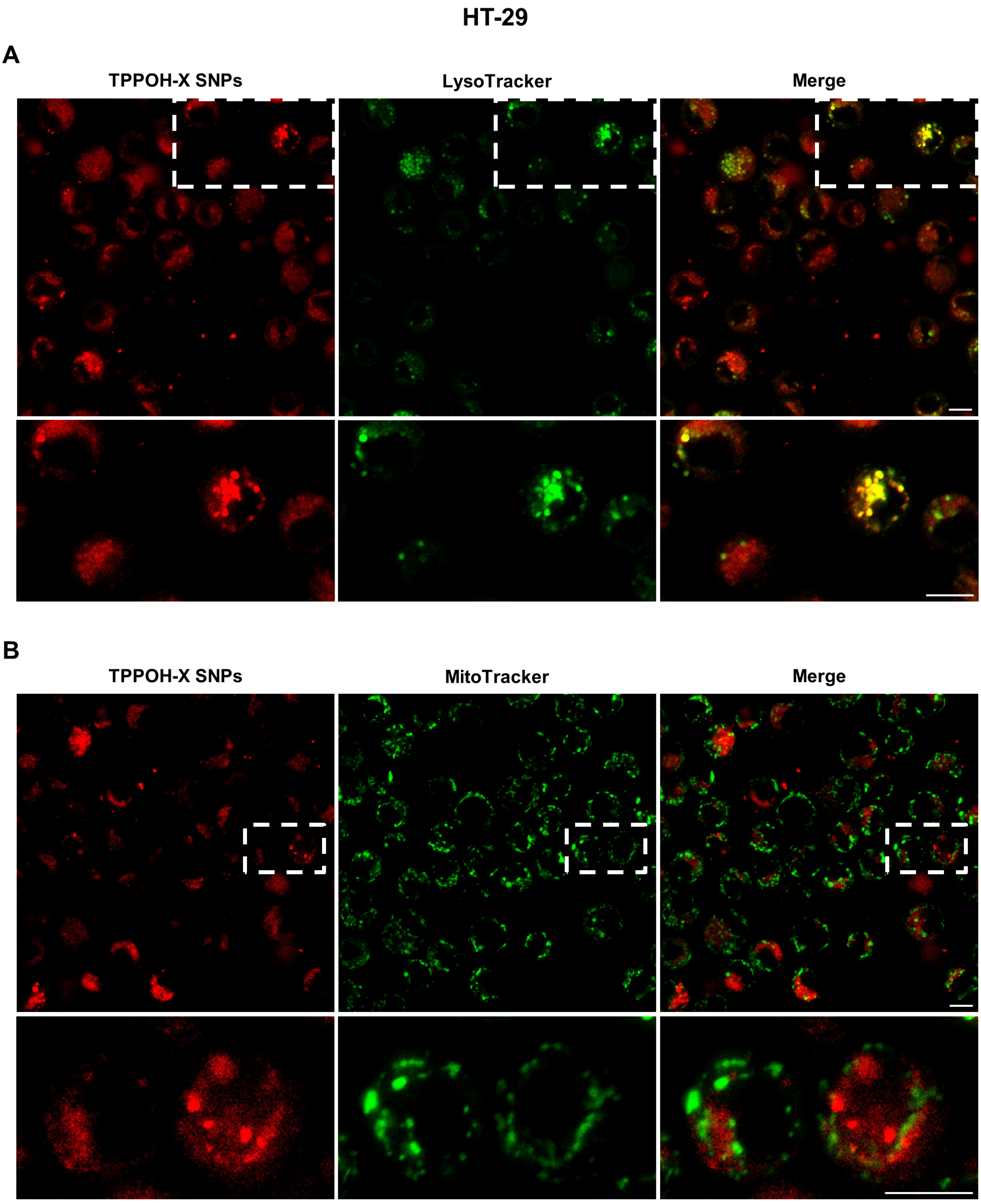
HT-29
A
TPPOH-X SNPs
LysoTracker
Merge
B
TPPOH-X SNPs
MitoTracker
Merge

Figure S5. TPPOH-X SNPs localization in HT-29 cells. (**A**) HT-29 cells were co-treated with TPPOH-X SNPs and LysoTracker or (**B**) MitoTracker and co-localization was studied 24 h post-treatment by confocal microscopy. Representative images of co-localization of TPPOH-X SNPs with LysoTracker or MitoTracker in HT-29 cells are shown. White scale bar = 10 µm. Data are shown as three independent experiments.

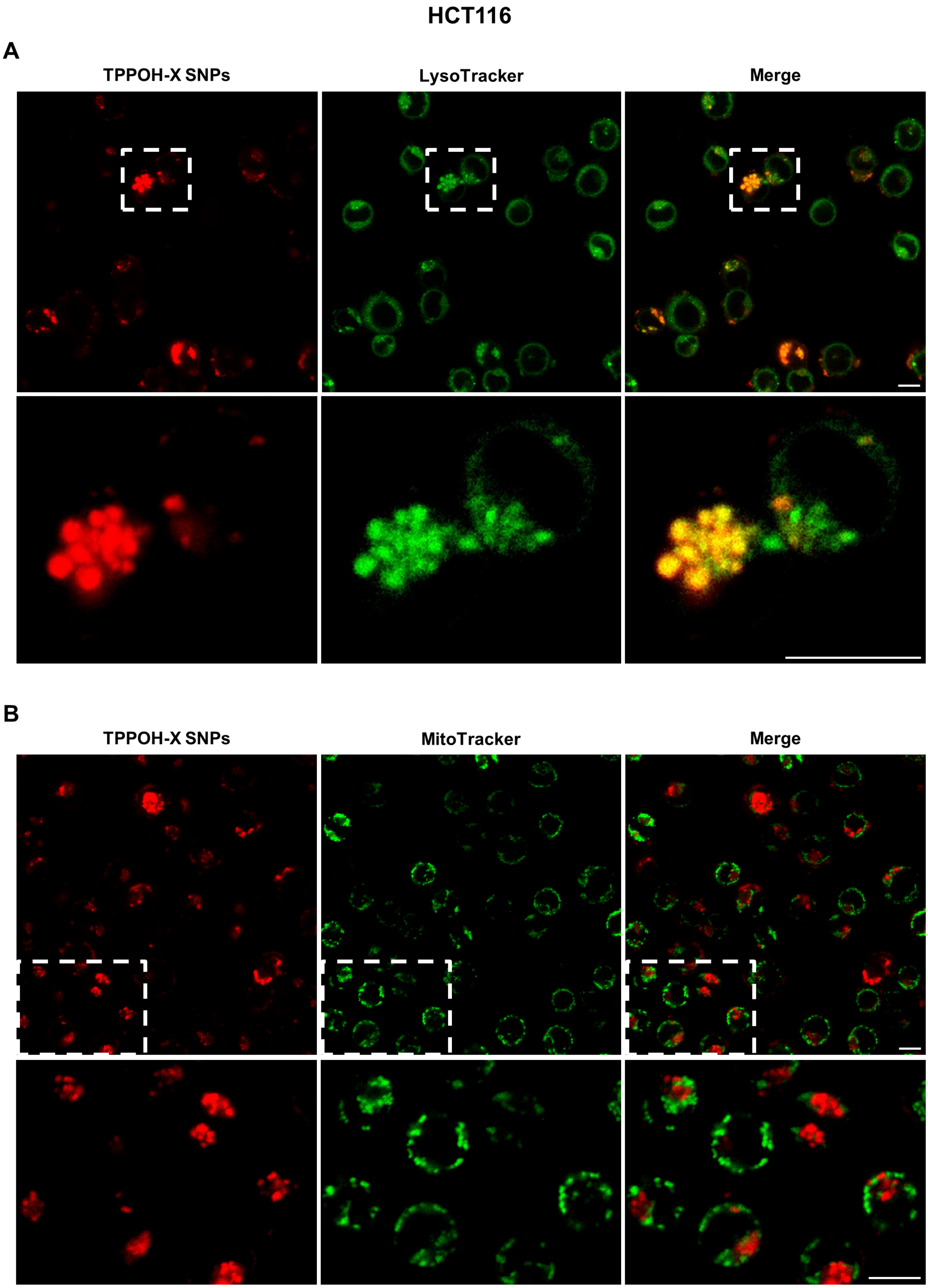
HCT116
A
TPPOH-X SNPs
LysoTracker
Merge
B
TPPOH-X SNPs
MitoTracker
Merge

Figure S6. TPPOH-X SNPs localization in HCT116 cells. (**A**) HCT116 cells were co-treated with TPPOH-X SNPs and LysoTracker or (**B**) MitoTracker and co-localization was studied 24 h post-treatment by confocal microscopy. Representative images of co-localization of TPPOH-X SNPs with LysoTracker or MitoTracker in HCT116 cells are shown. White scale bar = 10 µm. Data are shown as three independent experiments.

SW620

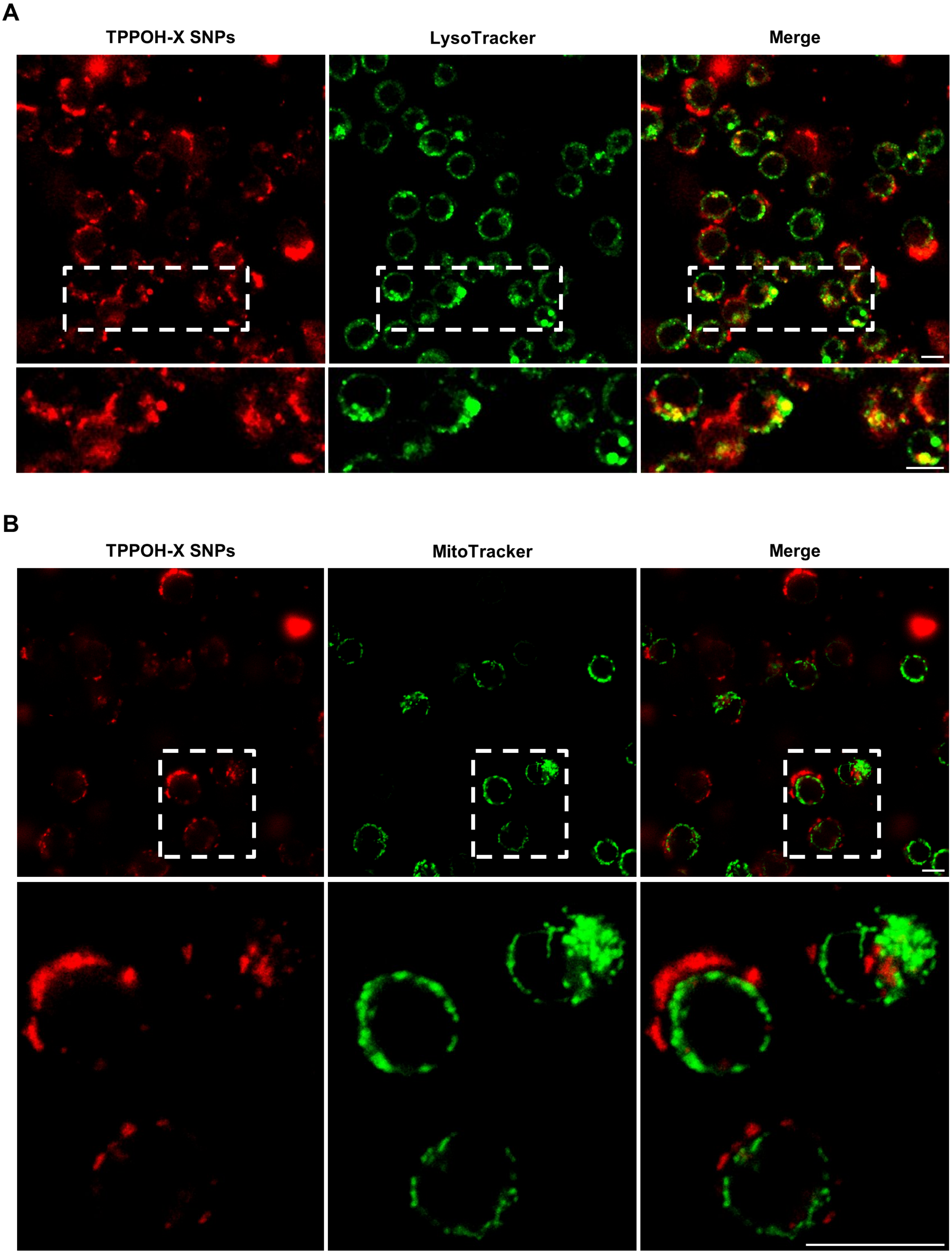
A
TPPOH-X SNPs
LysoTracker
Merge
B
TPPOH-X SNPs
MitoTracker
Merge

Figure S7. TPPOH-X SNPs localization in SW620 cells. (**A**) SW620 cells were co-treated with TPPOH-X SNPs and LysoTracker or (**B**) MitoTracker and co-localization was studied 24 h post-treatment by confocal microscopy. Representative images of co-localization of TPPOH-X SNPs with LysoTracker or MitoTracker in SW620 cells are shown. White scale bar = 10 μm. Data are shown as three independent experiments.

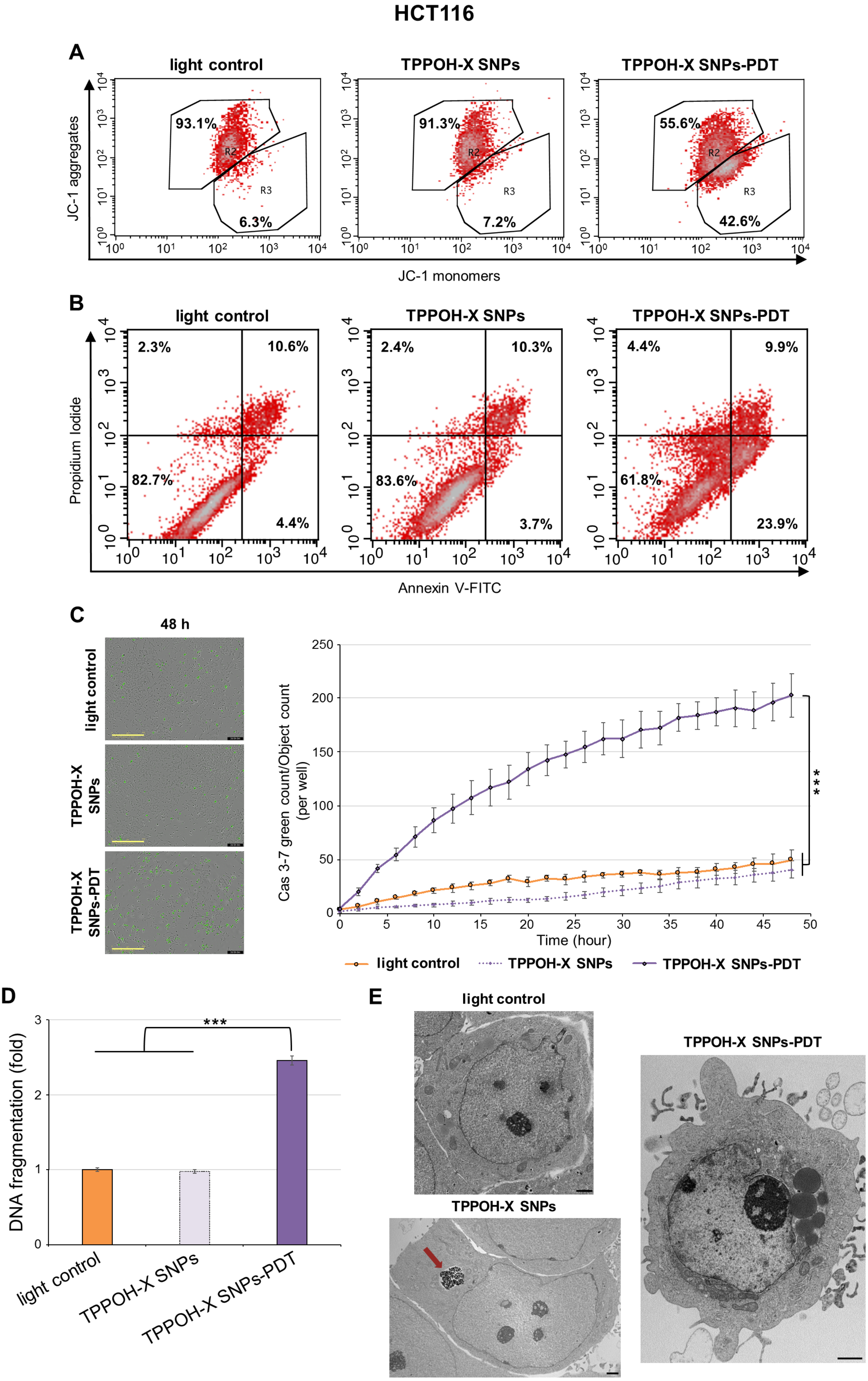

HCT116
A
light control
TPPOH-X SNPs
TPPOH-X SNPs-PDT
93.1%
6.3%
91.3%
7.2%
55.6%
42.6%
R2
R3
JC-1 aggregates
JC-1 monomers
B
light control
TPPOH-X SNPs
TPPOH-X SNPs-PDT
2.3%
10.6%
82.7%
4.4%
2.4%
10.3%
83.6%
3.7%
4.4%
9.9%
61.8%
23.9%
Propidium Iodide
Annexin V-FITC
C
48 h
light control
TPPOH-X SNPs
TPPOH-X SNPs-PDT
Cas 3-7 green count/Object count (per well)
Time (hour)
***
light control
TPPOH-X SNPs
TPPOH-X SNPs-PDT
D
DNA fragmentation (fold)
***
light control
TPPOH-X SNPs
TPPOH-X SNPs-PDT
E
light control
TPPOH-X SNPs
TPPOH-X SNPs-PDT

Figure S8. Effects of TPPOH-X SNPs-PDT on HCT116 cell line apoptosis. (**A**) HCT116 cells were treated or not with TPPOH-X SNPs and then photoactivated or not. The mitochondrial membrane potential was analyzed by flow cytometry with JC-1 at 48 h post-PDT. R2 represents the aggregate ratio and R3 the monomer ratio. (**B**) HCT116 cells were also stained, 48 h post-PDT, with Annexin V-FITC and PI, and apoptosis was analyzed by flow cytometry. Upper right quadrant represents the percentage of late apoptosis, and the lower right quadrant represents early apoptosis. (**C**) Caspase-3/7 activity, with the same conditions in HCT116 cells, was evaluated every 2 h during 48 h post-PDT by IncuCyte imaging live cell analysis and green count/cell count/well are shown. Representative images at 48 h post-PDT are shown. Yellow scale bar = 400 µm. (**D**) DNA fragmentation in HCT116 cells 48 h post-PDT was quantified from cytosol extracts by ELISA. Results are reported as n-fold compared to light control. (**E**) Representative TEM images of HCT116 cells treated or not with TPPOH-X SNPs and photoactivated or not 48 h post-PDT were shown. Red arrows indicate intracellular nanoparticles. Black scale bar = 1 µm. Data are shown as mean ± SEM (n = 3). $***p < 0.001$.

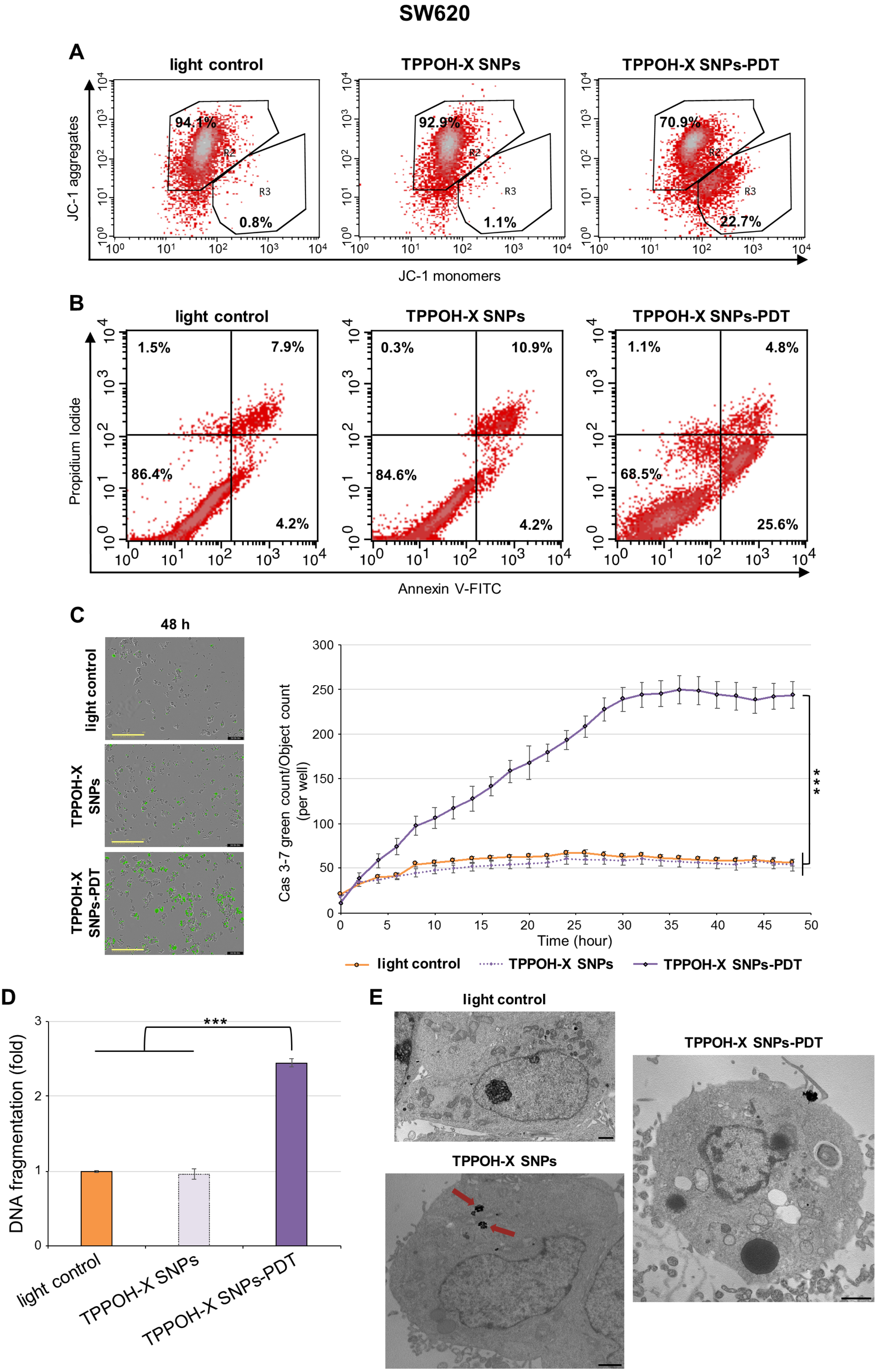
SW620
A
light control
TPPOH-X SNPs
TPPOH-X SNPs-PDT
JC-1 aggregates
JC-1 monomers
94.1%
0.8%
92.9%
1.1%
70.9%
22.7%
R2
R3
B
light control
TPPOH-X SNPs
TPPOH-X SNPs-PDT
Propidium Iodide
Annexin V-FITC
1.5%
7.9%
86.4%
4.2%
0.3%
10.9%
84.6%
4.2%
1.1%
4.8%
68.5%
25.6%
C
48 h
light control
TPPOH-X SNPs
TPPOH-X SNPs-PDT
Cas 3-7 green count/Object count (per well)
Time (hour)
***
light control
TPPOH-X SNPs
TPPOH-X SNPs-PDT
D
DNA fragmentation (fold)
***
light control
TPPOH-X SNPs
TPPOH-X SNPs-PDT
E
light control
TPPOH-X SNPs
TPPOH-X SNPs-PDT

Figure S9. Effects of TPPOH-X SNPs-PDT on SW620 cell line apoptosis. (**A**) SW620 cells were treated or not with TPPOH-X SNPs and then photoactivated or not. The mitochondrial membrane potential was analyzed by flow cytometry with JC-1 at 48 h post-PDT. R2 represents the aggregate ratio and R3 the monomer ratio. (**B**) SW620 cells were also stained, 48 h post-PDT, with Annexin V-FITC and PI, and apoptosis was analyzed by flow cytometry. Upper right quadrant represents the percentage of late apoptosis, and the lower right quadrant represents early apoptosis. (**C**) Caspase-3/7 activity, with the same conditions in SW620 cells, was evaluated every 2 h during 48 h post-PDT by IncuCyte imaging live cell analysis and green count/cell count/well are shown. Representative images at 48 h post-PDT are shown. Yellow scale bar = 400 µm. (**D**) DNA fragmentation in SW620 cells 48 h post-PDT was quantified from cytosol extracts by ELISA. Results are reported as n-fold compared to light control. (**E**) Representative TEM images of SW620 cells treated or not with TPPOH-X SNPs and photoactivated or not 48 h post-PDT were shown. Red arrows indicate intracellular nanoparticles. Black scale bar = 1 µm. Data are shown as mean ± SEM (n = 3). ***$p < 0.001$.

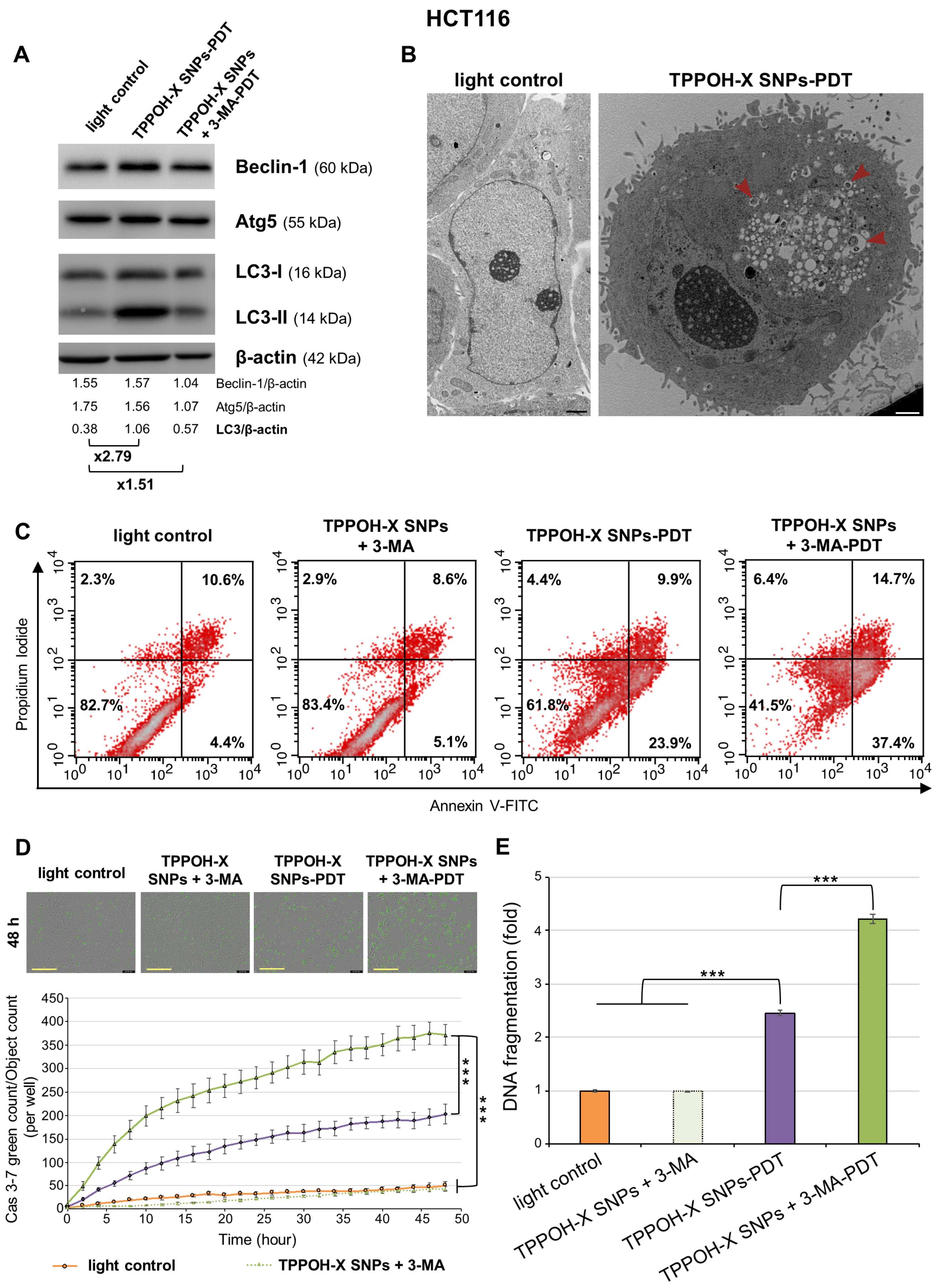

HCT116
A
light control
TPPOH-X SNPs-PDT
TPPOH-X SNPs + 3-MA-PDT
Beclin-1 (60 kDa)
Atg5 (55 kDa)
LC3-I (16 kDa)
LC3-II (14 kDa)
β-actin (42 kDa)
1.55 1.57 1.04 Beclin-1/β-actin
1.75 1.56 1.07 Atg5/β-actin
0.38 1.06 0.57 LC3/β-actin
x2.79
x1.51
B
light control
TPPOH-X SNPs-PDT
C
light control
TPPOH-X SNPs + 3-MA
TPPOH-X SNPs-PDT
TPPOH-X SNPs + 3-MA-PDT
Propidium Iodide
Annexin V-FITC
2.3% 10.6% 82.7% 4.4%
2.9% 8.6% 83.4% 5.1%
4.4% 9.9% 61.8% 23.9%
6.4% 14.7% 41.5% 37.4%
D
light control
TPPOH-X SNPs + 3-MA
TPPOH-X SNPs-PDT
TPPOH-X SNPs + 3-MA-PDT
48 h
Cas 3-7 green count/Object count (per well)
Time (hour)
***
***
light control
TPPOH-X SNPs + 3-MA
TPPOH-X SNPs-PDT
TPPOH-X SNPs + 3-MA-PDT
E
DNA fragmentation (fold)
***
***
light control
TPPOH-X SNPs + 3-MA
TPPOH-X SNPs-PDT
TPPOH-X SNPs + 3-MA-PDT

Figure S10. Effects of autophagy inhibition on HCT116 apoptosis. (**A**) HCT116 cells were treated or not with TPPOH-X SNPs in the presence or absence of 3-MA for 24 h. Expression of autophagy-related proteins was analyzed by Western blotting 48 h post-PDT. β-actin was used as a loading control. Representative images were shown. (**B**) Representative TEM images of HCT116 cells treated or not with TPPOH-X SNPs 48 h post-PDT protocol are shown. Red arrowheads indicate autophagosomes in the treated cells. Scale bar = 1 µm. (**C**) HCT116 cells were treated or not with TPPOH-X SNPs with or without 3-MA co-treatment and then photoactivated or not. At 48 h post-PDT, cells were stained with Annexin V-FITC and PI, and apoptosis was analyzed by flow cytometry. Upper right quadrant represents the percentage of late apoptosis, and the lower right quadrant represents early apoptosis. (**D**) With the same conditions of treatment, caspase-3/7 activity was evaluated each 2 h during 48 h post-PDT protocol by IncuCyte imaging live cell analysis and green count/cell count/well were shown. Representative images at 48 h post-PDT protocol were shown. Yellow scale bar = 400 µm. (**E**) With the same conditions of treatment, DNA fragmentation was quantified from cytosol extracts with ELISA. Results were reported as n-fold compared to light control. Data are shown as mean ± SEM (n = 3). ***$p < 0.001$.

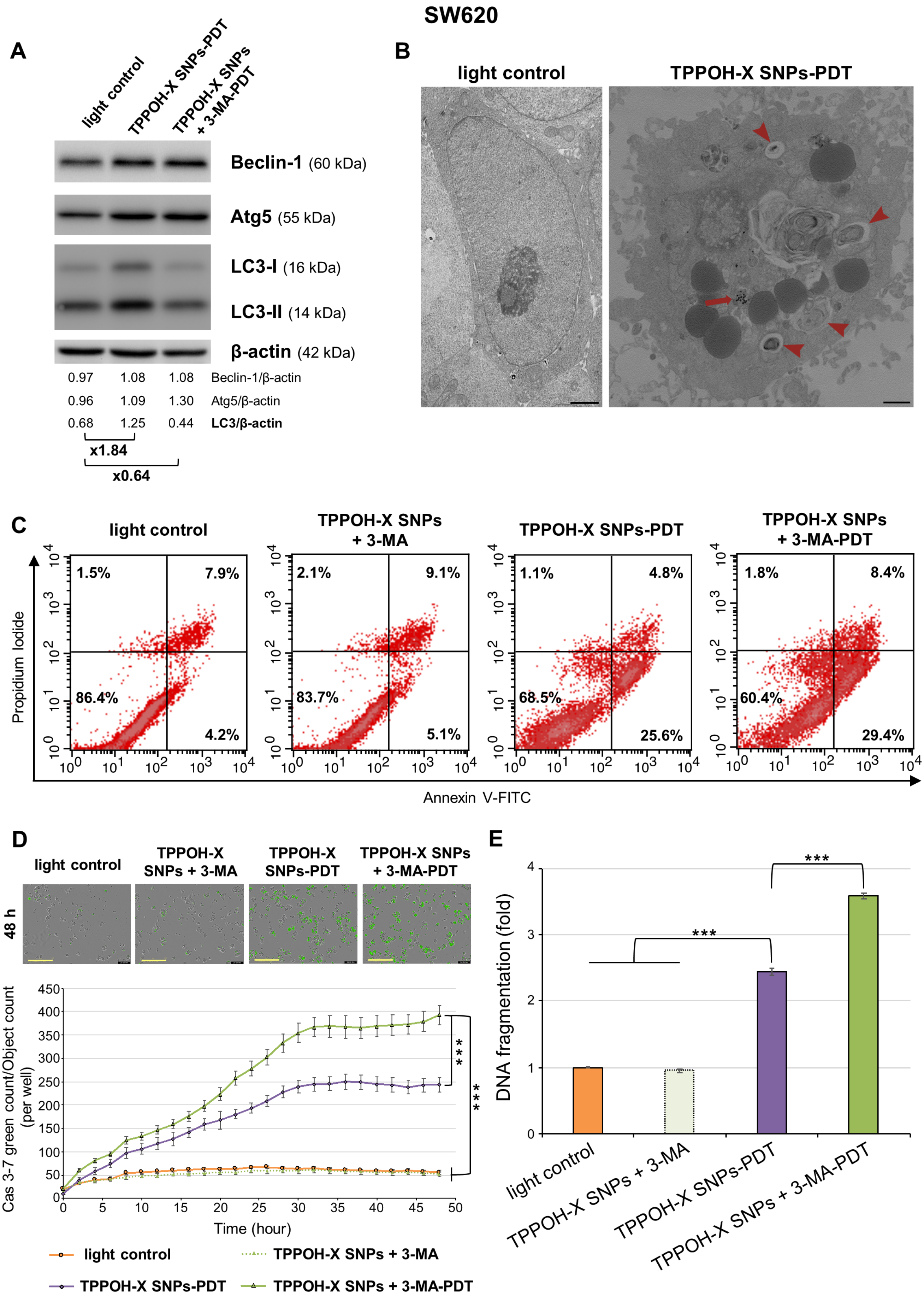
SW620
A
light control
TPPOH-X SNPs-PDT
TPPOH-X SNPs + 3-MA-PDT
Beclin-1 (60 kDa)
Atg5 (55 kDa)
LC3-I (16 kDa)
LC3-II (14 kDa)
β-actin (42 kDa)
0.97 1.08 1.08 Beclin-1/β-actin
0.96 1.09 1.30 Atg5/β-actin
0.68 1.25 0.44 LC3/β-actin
x1.84
x0.64
B
light control
TPPOH-X SNPs-PDT
C
light control
TPPOH-X SNPs + 3-MA
TPPOH-X SNPs-PDT
TPPOH-X SNPs + 3-MA-PDT
Propidium Iodide
1.5% 7.9% 86.4% 4.2%
2.1% 9.1% 83.7% 5.1%
1.1% 4.8% 68.5% 25.6%
1.8% 8.4% 60.4% 29.4%
Annexin V-FITC
D
light control
TPPOH-X SNPs + 3-MA
TPPOH-X SNPs-PDT
TPPOH-X SNPs + 3-MA-PDT
48 h
Cas 3-7 green count/Object count (per well)
Time (hour)
***
***
light control
TPPOH-X SNPs + 3-MA
TPPOH-X SNPs-PDT
TPPOH-X SNPs + 3-MA-PDT
E
DNA fragmentation (fold)
***
***
light control
TPPOH-X SNPs + 3-MA
TPPOH-X SNPs-PDT
TPPOH-X SNPs + 3-MA-PDT

Figure S11. Effects of autophagy inhibition on SW620 apoptosis. (**A**) SW620 cells were treated or not with TPPOH-X SNPs in the presence or absence of 3-MA for 24 h. Expression of autophagy-related proteins was analyzed by Western blotting 48 h post-PDT. β-actin was used as a loading control. Representative images were shown. (**B**) Representative TEM images of SW620 cells treated or not with TPPOH-X SNPs 48 h post-PDT protocol are shown. Red arrowheads indicate autophagosomes in the treated cells. Scale bar = 1 μm. (**C**) SW620 cells were treated or not with TPPOH-X SNPs with or without 3-MA co-treatment and then photoactivated or not. At 48 h post-PDT, cells were stained with Annexin V-FITC and PI, and apoptosis was analyzed by flow cytometry. Upper right quadrant represents the percentage of late apoptosis, and the lower right quadrant represents early apoptosis. (**D**) With the same conditions of treatment, caspase-3/7 activity was evaluated each 2 h during 48 h post-PDT protocol by IncuCyte imaging live cell analysis and green count/cell count/well were shown. Representative images at 48 h post-PDT protocol were shown. Yellow scale bar = 400 μm. (**E**) With the same conditions of treatment, DNA fragmentation was quantified from cytosol extracts with ELISA. Results were reported as n-fold compared to light control. Data are shown as mean ± SEM (n = 3). ***$p < 0.001$.

A

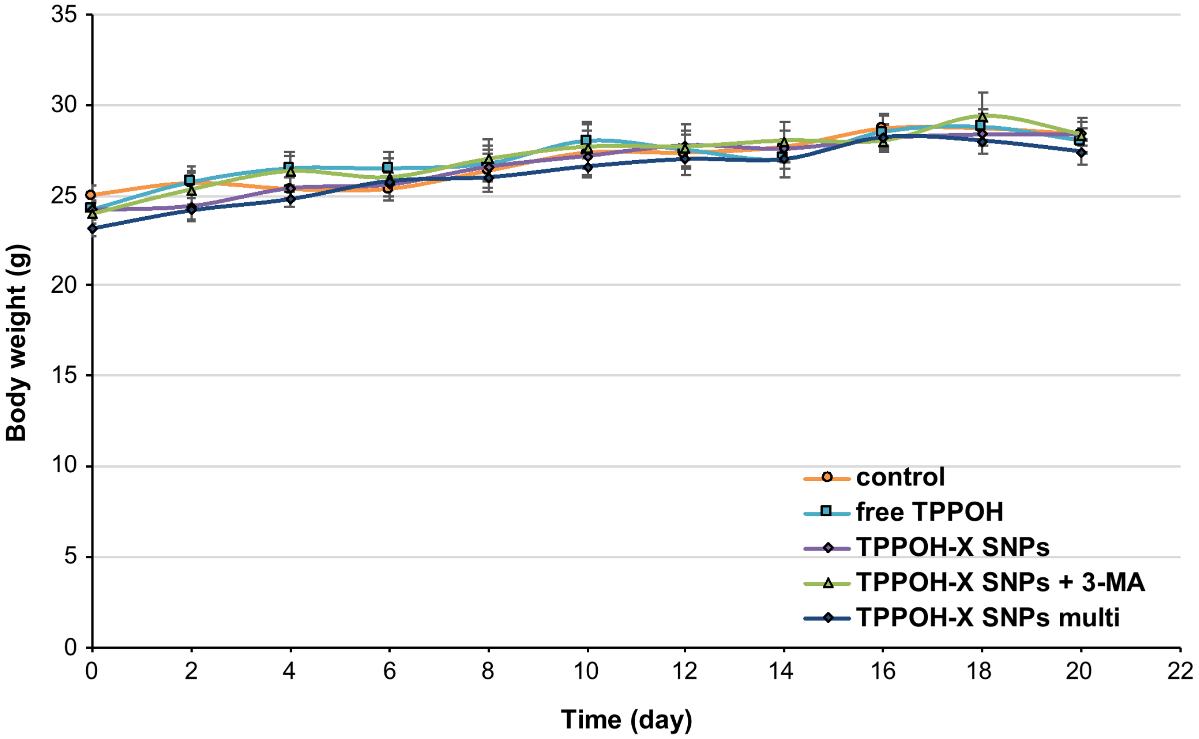

Body weight (g)
Time (day)
control
free TPPOH
TPPOH-X SNPs
TPPOH-X SNPs + 3-MA
TPPOH-X SNPs multi

B

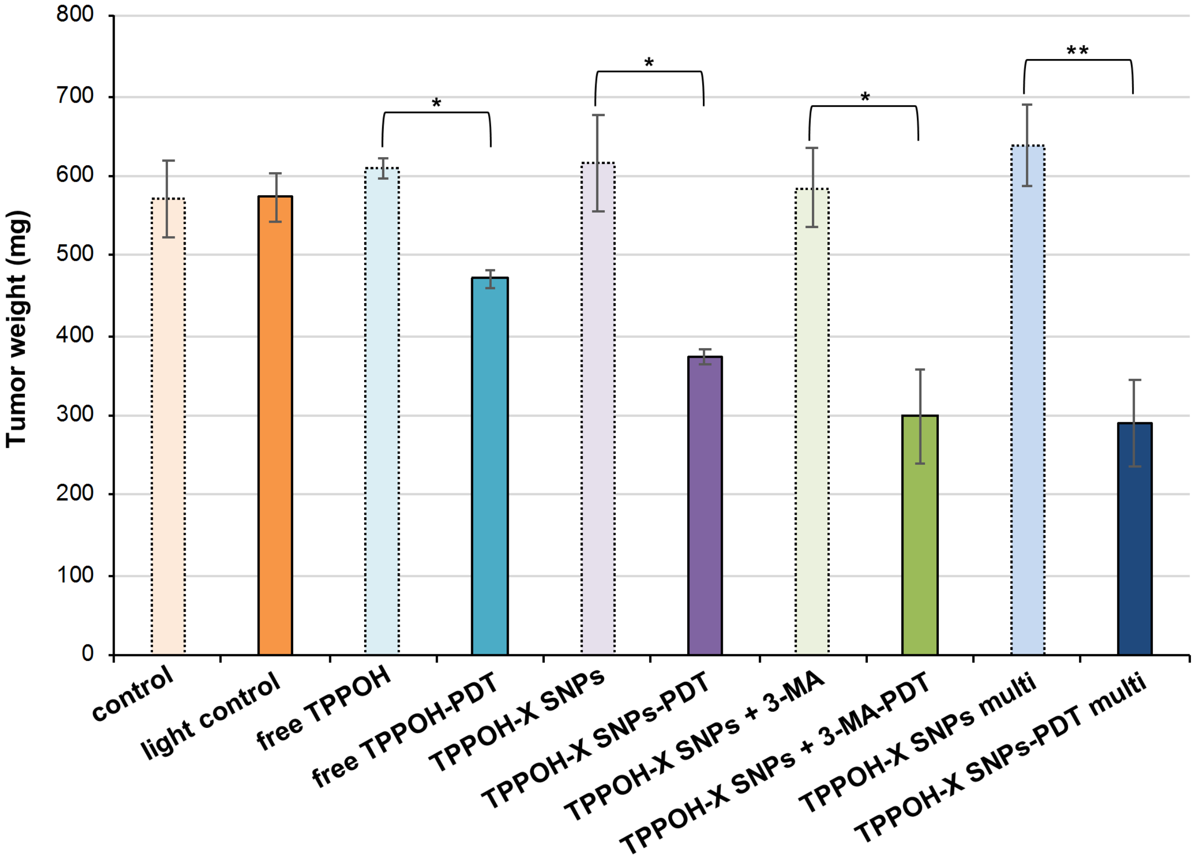

Tumor weight (mg)
control
light control
free TPPOH
free TPPOH-PDT
TPPOH-X SNPs
TPPOH-X SNPs-PDT
TPPOH-X SNPs + 3-MA
TPPOH-X SNPs + 3-MA-PDT
TPPOH-X SNPs multi
TPPOH-X SNPs-PDT multi

Figure S12. *In vivo* phototoxic effects on tumor growth. (**A**) Body weight variations of HT-29 tumor-bearing nude mice over the treatment period. (**B**) Tumor weight of the treatment groups after mice sacrifice. Data are shown as mean ± SEM ($n = 5$). *$p < 0.05$ and **$p < 0.01$.
